# Ramalin Ameliorates Alzheimer's Disease Pathology by Targeting BACE1, HDAC6, and MAPK Pathways

**DOI:** 10.1002/mco2.70518

**Published:** 2026-01-01

**Authors:** Yongeun Cho, Jeongmi Lee, Bo Youn Choi, Jin‐Ho Yun, Sukmin Han, Seung Hyun Baek, Jinsu Park, Yoonsuk Cho, Hark Kyun Kim, Eunae Kim, Leon F. Palomera, Jeein Lim, Yeji Jeon, Jeonghyeong Im, Ju‐Mi Hong, Tai Kyoung Kim, Sung Hyun Kim, Joung Han Yim, Dong‐Gyu Jo

**Affiliations:** ^1^ School of Pharmacy Sungkyunkwan University Suwon Republic of Korea; ^2^ Department of Neuroscience, Graduate School Kyung Hee University Seoul Republic of Korea; ^3^ Division of Polar Life Sciences Korea Polar Research Institute Incheon Republic of Korea; ^4^ Department of Physiology, School of Medicine Kyung Hee University Seoul Republic of Korea; ^5^ Biomedical Institute for Convergence Sungkyunkwan University Suwon Republic of Korea; ^6^ Samsung Advanced Institute For Health Sciences & Technology (SAIHST) Sungkyunkwan University Seoul Republic of Korea; ^7^ Institute of Quantum Biophysics Sungkyunkwan University Suwon Republic of Korea

**Keywords:** Alzheimer's disease, BACE1, HDAC6, NLRP3 (NLR family pyrin domain containing 3) inflammasome, ramalin

## Abstract

Aberrant deposition of β‐amyloid (Aβ) and hyperphosphorylated tau, along with neuroinflammation, are key drivers of Alzheimer's disease (AD) pathology. Here, we identify ramalin, a natural antioxidant, as a promising therapeutic agent that alleviates AD pathology by modulating β‐site APP cleaving enzyme 1 (BACE1), histone deacetylase 6 (HDAC6), and the mitogen‐activated protein kinases (MAPK) pathway. Ramalin reduced BACE1 protein levels, independently of its transcription, translation, or enzymatic activity, an effect mediated by inhibition of HDAC6. Consistently, HDAC6 knockout similarly decreased BACE1 levels, highlighting HDAC6 as a key regulator of BACE1. Ramalin further suppressed neuroinflammatory responses by downregulating inducible nitric oxide synthase (iNOS) and the NLR family pyrin domain containing 3 (NLRP3) inflammasome. In AD mouse models, ramalin treatment significantly attenuated neuroinflammation, Aβ plaque burden, and tau hyperphosphorylation, while improving cognitive performance. Notably, ramalin reversed Aβ oligomer‐induced synaptic transmission impairment and restored synaptic vesicle recycling in hippocampal neurons. Transcriptomic analysis identified modulation of the MAPK pathway, with reduced phosphorylation of c‐Jun N‐terminal kinase (JNK) and extracellular signal‐regulated kinase (ERK) implicated in tau pathology. These findings establish ramalin as a disease‐modifying intervention that provides neuroprotection through concurrent regulation of BACE1, HDAC6, and MAPK signaling pathway. Collectively, our findings highlight ramalin as a compelling disease‐modifying candidate with the potential to drive a breakthrough approach targeting AD pathology.

## Graphical abstract

Ramalin alleviates Alzheimer's disease pathology by selectively inhibiting HDAC6, reducing BACE1 levels, and suppressing neuroinflammation through downregulation of the NLRP3 inflammasome and iNOS. It restores synaptic function impaired by Aβ toxicity and improves cognitive performance in AD mouse models, APP/PS1 and 3xTg‐AD. Additionally, ramalin modulates the MAPK signaling pathway, reducing tau phosphorylation by inhibiting JNK and ERK activation.

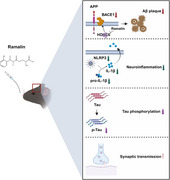

## 1 Introduction

Alzheimer's disease (AD) stands as the predominant cause of dementia in aging populations, marked by gradual decline in memory and cognition [1]. Global estimates suggest that dementia prevalence will continue to rise, with close to 150 million cases anticipated by 2050—an increase from roughly 57 million in 2019 [2]. In the United States, approximately 6 million individuals were affected by AD in 2020, and this number is forecast to rise to nearly 14 million by 2060, and the associated economic burden—estimated at about $307 billion in 2010—is expected to rise dramatically to more than $1.5 trillion by 2050 [3, 4]. Although numerous therapeutic approaches have been investigated, no disease‐modifying therapy is currently available for AD.

The pathological hallmarks of AD include the aberrant accumulation of β‐amyloid (Aβ) plaques, neurofibrillary tangles composed of hyperphosphorylated tau protein, neuroinflammation, and widespread neuronal loss. Aβ, a cleaved product of the amyloid precursor protein (APP), is generated predominantly in neurons as APP undergoes initial β‐secretase cleavage followed by γ‐secretase complex processing [5, 6, 7, 8]. This process gives rise to Aβ monomers, particularly Aβ_1–42_, which are prone to aggregation, forming toxic oligomers that impair dendritic spines and disrupt synaptic functions [9]. β‐site APP cleaving enzyme 1 (BACE1) is the key β‐secretase involved in Aβ production and is highly expressed in neurons and oligodendrocytes. BACE1‐mediated processing of APP is the rate‐determining step in Aβ production, thereby positioning BACE1 as a promising disease‐modifying target in AD [10, 11, 12, 13]. Even so, numerous BACE1 inhibitors have failed to demonstrate clinical efficacy, primarily due to adverse effects arising from the inhibition of multiple physiological substrates of BACE1, such as neuregulin‐1 and close homolog of L1 [14, 15]. Therefore, regulating BACE1 expression and activity may be a more viable therapeutic strategy than direct inhibition of the enzyme [16, 17, 18].

In addition to Aβ accumulation, neuroinflammation is now recognized as a critical contributor to AD progression. The NLR family pyrin domain containing 3 (NLRP3) inflammasome, a key mediator of the innate immune response, is activated in AD, promoting the production of inflammatory cytokines such as IL‐1β, which has been reported to be elevated in the plasma from individuals with AD [19, 20]. In AD, Aβ and tau aggregates act as danger cues that stimulate NLRP3 inflammasome activation in microglia, thereby perpetuating a cycle of inflammation and neurodegeneration [21, 22]. Recent studies have shown that pharmacologically blocking or genetically ablating NLRP3 can attenuate Aβ deposition and cognitive deficits in preclinical models of AD [20, 23].

Histone deacetylases (HDACs) are enzymes crucial for the regulation of the epigenome through chromatin remodeling. These enzymes contribute to diverse cellular processes, including long‐term memory formation and synaptic plasticity, by modulating gene expression epigenetically [24, 25]. In line with these functions, HDACs are regarded as attractive therapeutic targets in cancer, neurodegeneration, vascular disease, and inflammatory states [26, 27]. Among them, HDAC6 has gained particular attention in the context of AD. HDAC6 is notably upregulated in the brains of AD patients and 5xFAD mice, implicating its involvement in AD pathogenesis [28, 29]. Recent studies have demonstrated that HDAC6 regulates tau phosphorylation and either genetic ablation or pharmacological inhibition of HDAC6 ameliorates cognitive deficits in AD models by reducing Aβ and tau pathology [30, 31]. Furthermore, HDAC6 appears to contribute to inflammasome activation by directing NLRP3 trafficking from the trans‐Golgi network to the microtubule‐organizing center, where the complex forms [32]. Pharmacological inhibition of HDAC6 has been shown to dampen NLRP3 inflammasome and consequently reduce IL‐1β levels [33].

Mitogen‐activated protein kinases (MAPKs) are a family of serine/threonine kinases that transmit intracellular signals regulating cellular processes such as proliferation and survival [34]. MAPK subfamilies comprise extracellular signal‐regulated kinase (ERK), c‐Jun N‐terminal kinase (JNK), and p38 MAPK. Aberrant MAPK pathway activation contributes to AD pathology by facilitating tau hyperphosphorylation [35]. JNK, ERK, and p38 MAPK phosphorylate tau at disease‐relevant sites, thereby promoting neurofibrillary tangle formation, synaptic dysfunction, and neuronal loss. Aberrant activation of these pathways accelerates disease progression, whereas pharmacological inhibition of JNK mitigates tau pathology [36, 37, 38].

Ramalin is a natural compound isolated from Antarctic lichen *Ramalina terebrata*, which has been reported to possess potent antioxidant and antibacterial properties [39, 40]. Additionally, it has shown anticancer activity by inhibiting the proliferation of colorectal cancer cells and inducing apoptosis and cell cycle arrest via modulation of tumor protein p53 [41]. More recently, ramalin has been regarded as an attractive therapeutic candidate for AD because it can mitigate neuroinflammation by reducing nitric oxide (NO) generation induced by lipopolysaccharide (LPS) stimulation [42].

In this study, we explored ramalin as a therapeutic intervention for AD. Through both in vitro and in vivo models, we examined its molecular target and assessed its effects on Aβ production, neuroinflammatory responses, and cognitive function. Overall, ramalin exerted multifaceted effects across AD‐related pathways, reinforcing its therapeutic promise.

## 2 Results

### 2.1 Ramalin Reduces BACE1 Protein Expression without Affecting Transcription, Translation, or Activity

**FIGURE 1:**
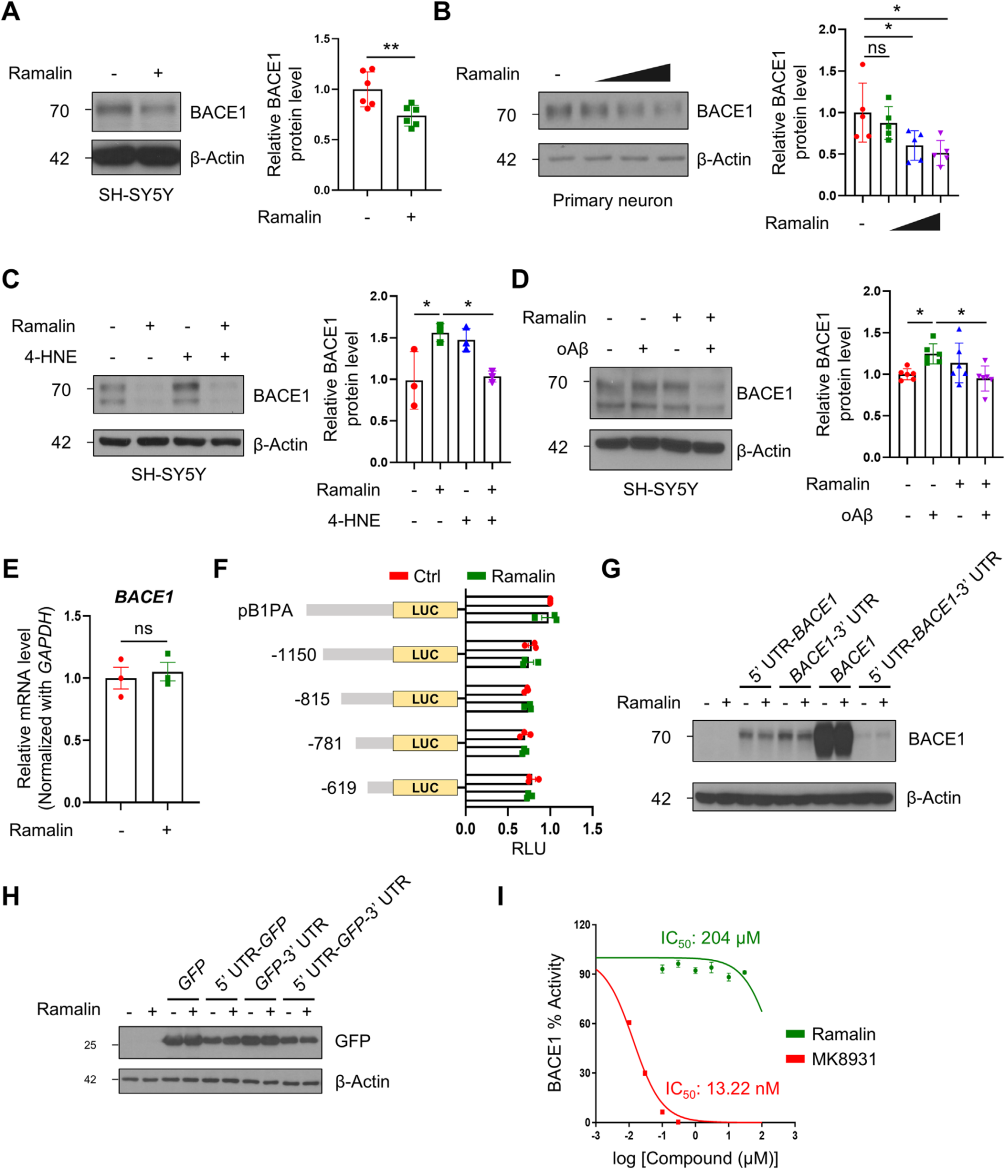
Ramalin lowers BACE1 protein levels without affecting transcription, translation, or BACE1 activity. (A) Western blot analysis and quantification of BACE1 protein levels in SH‐SY5Y cells treated with 1 µM of ramalin (*n *= 6 per group). (B) Western blot analysis and quantification of BACE1 protein levels in murine primary neurons (*n *= 5 per group). (C) Western blot analysis and quantification of BACE1 protein levels in SH‐SY5Y cells treated with 1 µg/mL of ramalin and 5 µM of 4‐HNE (*n *= 3 per group). (D) Western blot analysis and quantification of BACE1 protein levels in SH‐SY5Y cells treated with 5 µM of ramalin and 2 µM of oAβ (*n *= 6 per group). (E) RT‐qPCR analysis of *BACE1* mRNA levels in SH‐SY5Y cells in response to 5 µM of ramalin treatment. *BACE1* mRNA expression levels were normalized with *GAPDH* (*n *= 3). (F) *BACE1* promoter activity was measured through luciferase assay in HEK293T cells transfected with pB1PA and *BACE1* promoter deletion constructs and then treated with 10 µM of ramalin (*n* = 3). (G) Western blot analysis of BACE1 protein levels in *Bace1* KO MEF cells transfected with the UTR of *BACE1* and treated with 1 µM of ramalin. (H) Western blot analysis of GFP protein levels in HEK293T cells transfected with the UTR of *BACE1* and treated with 1 µM of ramalin. (I) BACE1 enzyme activity assay was conducted using ramalin and MK8931, a potent BACE1 inhibitor used as a positive control. The IC_50_ of ramalin on BACE1 was determined to be 204 µM. Data are shown as mean ± SD in (A), (B), (C), (D) or mean ± SEM in (E), (F), and (I). Data were analyzed using one‐way ANOVA followed by Dunnett's test in (B), (C), (D) or unpaired two‐tailed *t*‐test in (A), (E), and (F). ns; *p* > 0.05; *p < 0.05; **p < 0.01.

To investigate the effects of ramalin on regulating BACE1 expression, we treated both SH‐SY5Y neuroblastoma cells and mouse primary cortical neurons with increasing concentrations of ramalin. In both cell types, we observed a significant and dose‐dependent reduction in BACE1 protein levels (Figure 1A, B). Notably, ramalin mitigated the upregulation of BACE1 induced by 4‐hydroxynonenal (4‐HNE), a lipid peroxidation product known to exacerbate oxidative stress and increase BACE1 expression (Figure 1C) [43, 44]. Moreover, ramalin effectively reduced BACE1 expression in response to stimulation with oligomeric Aβ (Figure 1D).

To determine whether ramalin modulates *BACE1* expression at the transcriptional level, we assessed *BACE1* mRNA levels and promoter activity using a luciferase reporter assay. Our data revealed that neither BACE1 mRNA expression nor promoter activity was altered by ramalin treatment (Figure 1E, F), indicating that the reduction in BACE1 protein levels is not mediated by changes in transcriptional regulation.

Given the lack of transcriptional effects, we next examined whether ramalin affects posttranscriptional mechanisms, specifically the regulation of *BACE1* mRNA through its untranslated regions (UTRs). We generated constructs containing the 5’ UTR and/or 3′ UTR of *BACE1* and transfected these into *Bace1* knockout mouse embryonic fibroblast (Bace1 KO‐MEF) cells. Ramalin reduces BACE1 protein levels independent of the presence of the 5′ or 3′ UTR of *BACE1* (Figure 1G). To validate ramalin did not affect UTRs of *BACE1*, we generated GFP constructs containing UTRs of *BACE1* and transfected these into HEK293T cells. Ramalin had no effects on GFP levels in UTR‐containing constructs, suggesting that ramalin does not modulate BACE1 expression via mRNA stability or translational control (Figure 1H).

We then examined whether ramalin inhibits the proteolytic activity of BACE1 using MK8931, a potent BACE1 inhibitor, as a positive control. Ramalin did not reduce BACE1 activity to the same extent as MK8931 (Figure 1I), suggesting that ramalin does not act as a direct BACE1 protease inhibitor.

### 2.2 Ramalin does not Affect BACE1 Degradation via the Ubiquitin–Proteasome System or Autophagy

To determine whether ramalin modulates BACE1 protein degradation, we investigated its effects on both the ubiquitin–proteasome system (UPS) and autophagic pathways which are two primary mechanisms for protein turnover. We first evaluated the involvement of the UPS by fusing the CL1 degron sequence, known for targeting proteins to rapid proteasomal degradation, to GFP (Figure S1A) [45]. Upon transfection into HEK293T cells, ramalin treatment did not alter GFP expression levels, indicating that ramalin does not enhance the proteasomal degradation of BACE1 (Figure S1B).

Next, we investigated the role of autophagy in ramalin‐mediated BACE1 regulation. Using chloroquine (CQ), an autophagy inhibitor, we blocked autophagic flux in cells treated with ramalin. BACE1 protein levels remained unchanged in the presence of both CQ and ramalin (Figure S1C), suggesting that autophagic degradation is not a mechanism through which ramalin exerts its effects on BACE1 expression.

Taken together, these results indicate that ramalin does not influence BACE1 degradation through either the UPS or autophagy, further supporting the hypothesis that ramalin regulates BACE1 through alternative mechanisms that do not involve conventional protein degradation pathways.

### 2.3 Identification of HDAC6 as a Putative Target of Ramalin in BACE1 Regulation

**FIGURE 2:**
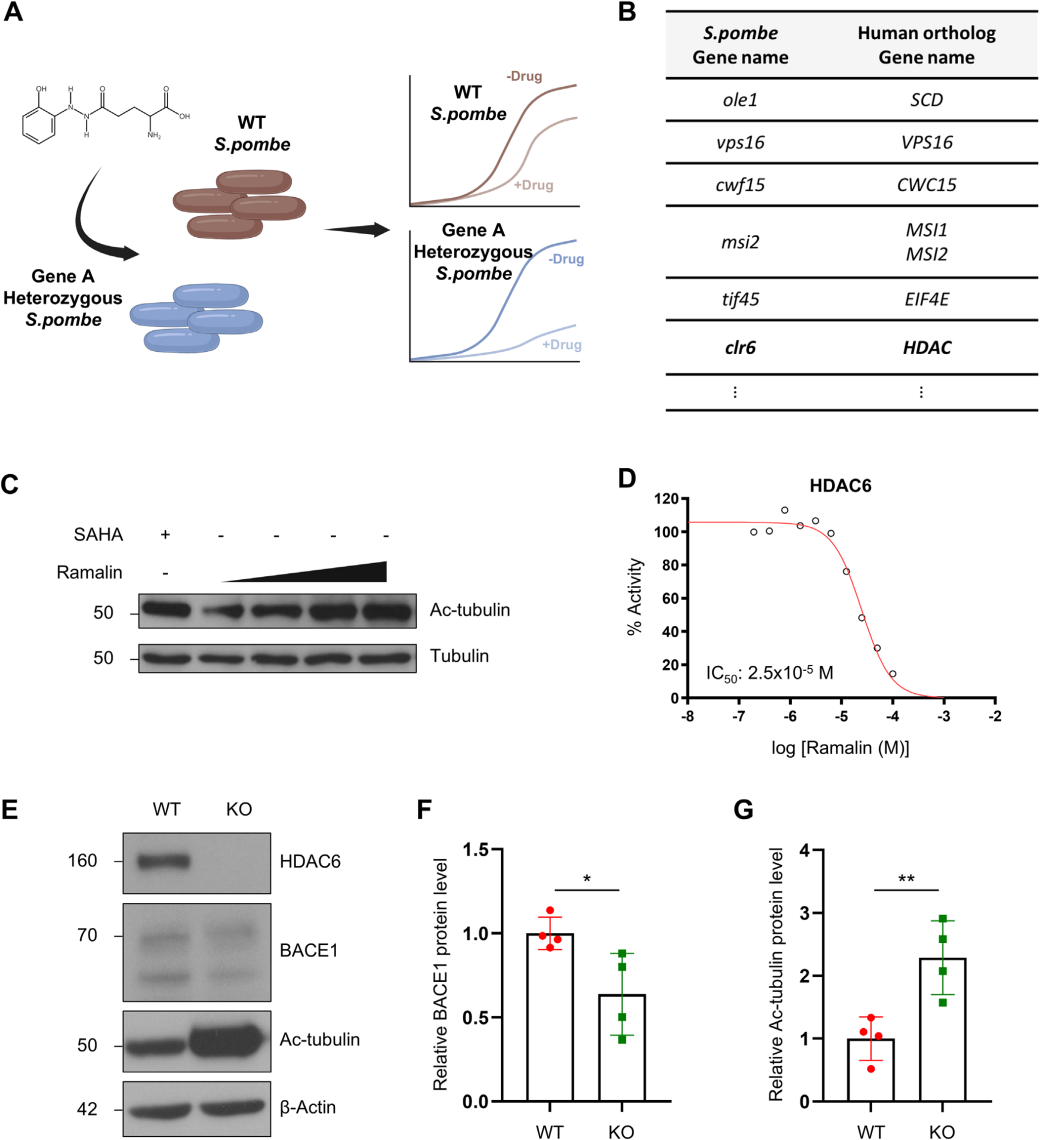
Ramalin modulates BACE1 levels through HDAC6 inhibition. (A) Schematic diagram of *GPScreen* ^TM^. The figure was created with Biorender.com. (B) List of predicted *S.pombe* target genes and its human ortholog gene of ramalin identified by *GPScreen* ^TM^. (C) Western blot analysis of acetylated tubulin (Ac‐tubulin) and tubulin protein levels in SH‐SY5Y cells treated with 5–15 µM of ramalin and 0.1 µM of suberoylanilide hydroxamic acid (SAHA). SAHA was used as a positive control. (D) IC_50_ value of ramalin on HDAC6 was presumed to be 25 µM identified by HDAC6 inhibitory assay. (E) Western blot analysis of HDAC6, BACE1, and Ac‐tubulin protein levels in WT SH‐SY5Y and HDAC6 KO SH‐SY5Y. (F) Quantification of BACE1 protein levels in (E) (*n* = 4). The expression level of BACE1 was normalized to the expression level of β‐actin. (G) Quantification of Ac‐tubulin protein levels in (E) (*n *= 4). The expression level of Ac‐tubulin was normalized to the expression level of β‐actin. Data are shown as mean ± SD in (F) and (G). Data were analyzed using unpaired two‐tailed *t*‐test in (F) and (G). *p < 0.05; **p < 0.01.

To identify the molecular targets of ramalin that might contribute to its regulatory effect on BACE1, we conducted a genome‐wide *GPScreen* assay. *GPScreen* assay measures drug‐induced haploinsufficiency (DIH) in *S.pombe* gene deletion mutant library to reveal potential drug target (Figure 2A) [46, 47]. Several potential targets were identified, including stearoyl‐CoA desaturase (SCD), vacuolar protein sorting‐associated protein 16 (VPS16), CWC15 spliceosome‐associated protein homolog (CWC15), Musashi RNA‐binding protein 1, and 2 (MSI1 and MSI2), eukaryotic translation initiation factor 4E (EIF4E), and HDACs (Figure 2B). Initial experiments in SH‐SY5Y cells with MK8245, a selective SCD inhibitor, did not reduce BACE1 levels (Figure S2A). Similarly, siRNA‐mediated knockdown of *VPS16*, *CWC15*, *MSI1*, *MSI2*, and *EIF4E* in HEK293T cells did not affect BACE1 expression (Figure S2B, D).

Next, we examined whether ramalin exerts its effect on BACE1 through HDAC modulation. Ramalin dose‐dependently increased tubulin acetylation in SH‐SY5Y cells, where tubulin is known as a target of HDAC6 (Figure 2C). However, treatment with pan‐HDAC inhibitors, such as SAHA and phenylbutyrate, did not reduce BACE1 levels (Figure S3A, B). HDACs assays further revealed that ramalin selectively inhibits HDAC6 (IC_50_ = 2.5 × 10^−5^ M) over other zinc dependent HDAC members (Figures 2D and S4). Ramalin did not inhibit NAD^+^‐dependent HDACs (Figure S5).

Notably, CRISPR–Cas9‐mediated knockout of HDAC6 significantly decreased BACE1 protein levels and increased Ac‐tubulin protein levels in SH‐SY5Y cells (Figure 2E–G). This suggests that the ability of ramalin to decrease BACE1 expression may be mediated through selective inhibition of HDAC6, rather than broad HDAC inhibition. These findings position HDAC6 as a putative target of ramalin for BACE1 regulation and highlight a novel mechanism by which ramalin may exert its neuroprotective effects in AD.

### 2.4 Ramalin Exhibits Potent Anti‐Inflammatory Effects

**FIGURE 3:**
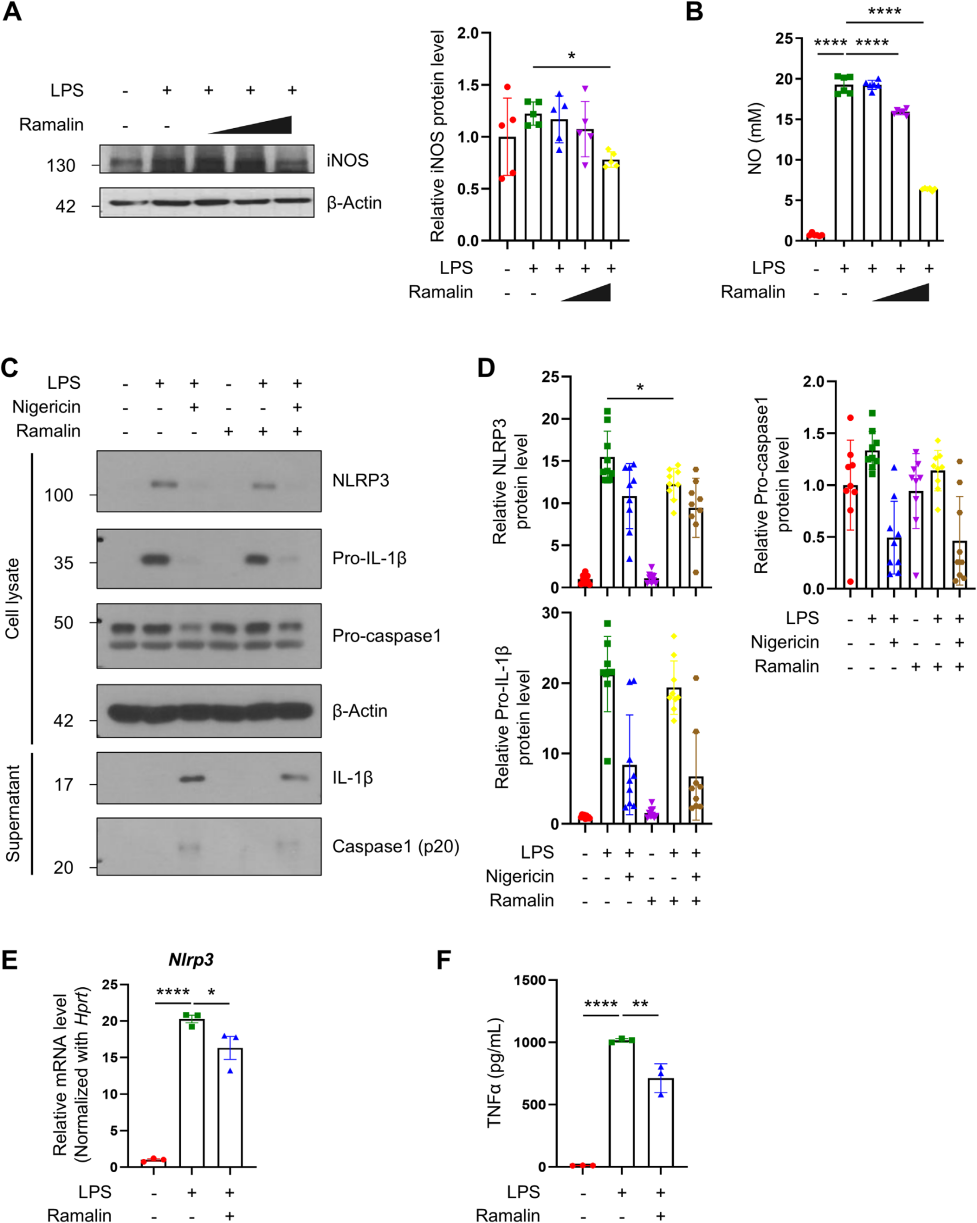
Ramalin shows protective effects against neuroinflammation in AD. (A) Western blot analysis and quantification of iNOS protein levels in BV‐2 cells treated with 0.1, 1, 10 µg/mL of ramalin for 30 min followed by exposure to 1 µg/mL of LPS for 24 h (*n *= 5 per group). (B) NO production in BV‐2 cells treated with 0.1, 1, 10 µg/mL of ramalin for 30 min followed by exposure to 1 µg/mL of LPS for 24 h (*n *= 6 per group). (C) Western blot analysis of NLRP3, IL‐1β and caspase‐1 protein levels in murine primary microglia treated with 20 µM of ramalin. 0.2 µg/mL of LPS was added for 6 h, followed by treatment with 20 µM of nigericin for 30 min. (D) Quantification of NLRP3, pro‐caspase‐1, and pro‐IL‐1β protein level in (C) (*n *= 9 per group). The expression level of NLRP3, pro‐caspase‐1, and pro‐IL‐1β was normalized to the expression level of β‐actin. (E) RT‐qPCR analysis of *Nlrp3* mRNA levels in BV‐2 cells treated with 5 µM of ramalin for 24 h followed by exposure to 0.2 µg/mL of LPS for 6 h (*n *= 3 per group). (F) TNFα production in THP‐1 cells treated with 10 µM of ramalin followed by 0.1 µg/mL of LPS treatment (*n* = 3 per group). Data are shown as mean ± SD in (A), (B), (D), (F) and as mean ± SEM in (E). Data were analyzed using one‐way ANOVA followed by Dunnett's test. *p < 0.05; **p < 0.01; ****p < 0.0001.

Considering the pivotal role of neuroinflammation in AD pathogenesis, we evaluated the anti‐inflammatory properties of ramalin, particularly its effects on inducible NO synthase (iNOS) and NO, both of which are implicated in AD‐related synaptic dysfunction and neuronal loss [48, 49]. We treated BV‐2 microglial cells with increasing concentrations of ramalin, followed by stimulation with LPS to induce an inflammatory response. Ramalin treatment showed a significant, dose‐dependent reduction in iNOS protein expression (Figure 3A) and concomitantly decreased NO production, as measured by the Griess assay (Figure 3B). These results suggest that ramalin effectively suppresses iNOS/NO‐mediated neuroinflammatory responses.

We next investigated the effects of ramalin on the NLRP3 inflammasome, a critical regulator of chronic inflammation in AD. Treatment of primary mouse microglia with ramalin significantly decreased the NLRP3 inflammasome signaling pathway, including NLRP3, active caspase‐1, and its product IL‐1β (Figure 3C, D). Furthermore, ramalin downregulated *Nlrp3* mRNA expression in LPS‐stimulated BV‐2 cells (Figure 3E). Additionally, we examined its effects on tumor necrosis factor‐alpha (TNF‐α), a proinflammatory cytokine involved in neurodegeneration. Ramalin significantly attenuated TNF‐α expression in LPS‐stimulated THP‐1 monocytes (Figure 3F).

These findings collectively demonstrate that ramalin exerts broad‐spectrum anti‐inflammatory effects by targeting multiple inflammatory pathways, including iNOS/NO, the NLRP3 inflammasome, and TNF‐α signaling.

### 2.5 Ramalin Prevents Synaptic Transmission Impairment Induced by Aβ Oligomers

**FIGURE 4:**
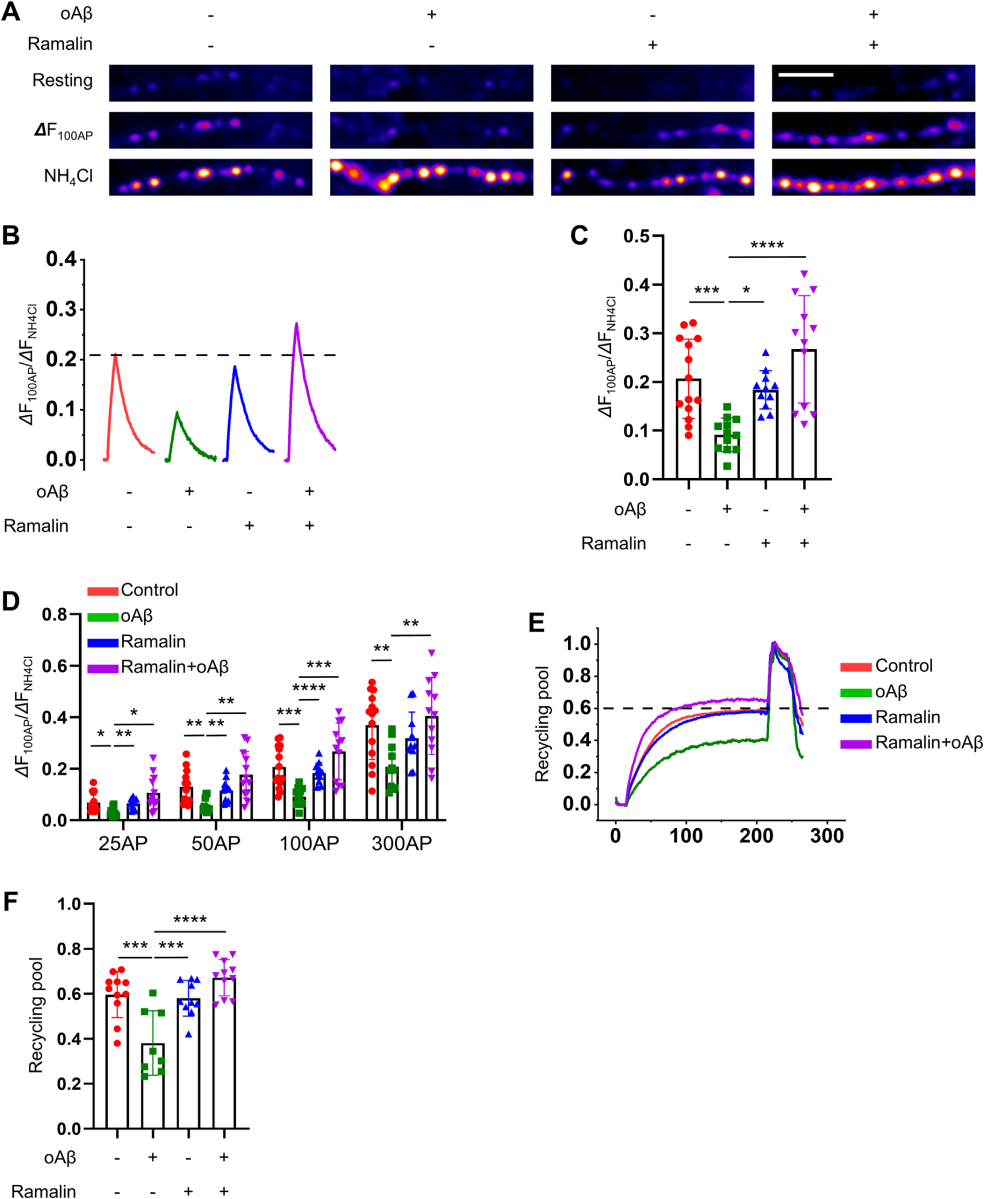
Ramalin prevents synaptic dysfunction in hippocampal neurons under pathologic condition. (A) Representative images of vG–pH in the condition of resting, 100 AP (DF_100AP_), and NH_4_Cl at synapses (scale bar = 5 mm). (B) Representative vG–pH ensemble‐averaged traces evoked by 100 AP in control (red), Aβ (green), ramalin (blue), and Aβ with ramalin (purple)‐treated neurons. vG–pH‐expressing neurons were subjected to 10 Hz stimulation for 10 s in the presence or absence of Aβ and ramalin. Fluorescence signals were normalized to the peak response induced by NH_4_Cl. (C) Mean amplitudes of 100 AP responses in control, Aβ, ramalin, and Aβ+ramalin‐treated neurons. [Con]_100AP_ = 0.21 ± 0.02 (*n *= 14); [Aβ]_100AP_ = 0.09 ± 0.01 (*n *= 12); [Ramalin]_100AP_ = 0.18 ± 0.01 (*n *= 11); [Aβ+Ramalin]_100AP_ = 0.27 ± 0.03 (*n *= 12). (D) Mean values of amplitudes of various stimulations in control, Aβ, ramalin, and Aβ+ramalin‐treated neurons. [Con]_25AP_ = 0.07 ± 0.01 (*n *= 14); [Aβ]_25AP_ = 0.03 ± 0.005 (*n *= 11); [Ramalin]_25AP_ = 0.06 ± 0.01 (*n *= 11); [Aβ+Ramalin]_25AP_ = 0.11 ± 0.02 (*n *= 12), [Con]_50AP_ = 0.13 ± 0.02 (*n *= 14); [Aβ]_50AP_ = 0.06 ± 0.01 (*n *= 11); [Ramalin]_50AP_ = 0.12 ± 0.01 (*n *= 11); [Aβ+Ramalin]_50AP_ = 0.18 ± 0.03 (*n *= 12), [Con]_300AP_ = 0.37 ± 0.04 (*n *= 14); [Aβ]_300AP_ = 0.21 ± 0.03 (*n *= 11); [Ramalin]_300AP_ = 0.32 ± 0.03 (*n *= 11); [Aβ+Ramalin]_300AP_ = 0.40 ± 0.04 (*n *= 12). (E) Representative ensemble traces of vG–pH in response to 2,000 AP in the presence of bafilomycin in control (red), Aβ (green), ramalin (blue), and Aβ+ramalin (purple)‐treated neurons. (F) Mean values of recycling vesicle pool size in responses to 2,000 AP at 10 Hz in the presence of bafilomycin A1 (BAF) in control (0.60 ± 0.03, *n *= 11), Aβ (0.38 ± 0.05, *n *= 8), ramalin (0.58 ± 0.03, *n *= 10), and Aβ+ramalin (0.67 ± 0.02, *n *= 11). Data are shown as mean ± SEM in (C), (D), and (F). Data were analyzed using one‐way ANOVA followed by Tukey's test in (C) and (F) and two‐way ANOVA followed by Tukey's test in (D). *p < 0.05; **p < 0.01; ***p < 0.001; ****p < 0.0001.

Synaptic dysfunction represents one of the major pathological features observed in AD [50], and is exacerbated by the accumulation of toxic Aβ oligomers. To assess whether ramalin could protect synaptic function, we employed a pHluorin‐based assay to directly measure synaptic vesicle recycling and neurotransmitter release in primary mouse hippocampal neurons. pHluorin is fused to the luminal domain of the vesicular glutamate transporter (vGlut1–pHluorin [vG–pH]) [51]. Under normal conditions, ramalin treatment did not alter synaptic transmission (Figure 4A–D). However, in pathological conditions where neurons were exposed to Aβ oligomers, synaptic transmission was significantly impaired, decreasing to approximately 9% of normal levels. Remarkably, cotreatment with ramalin substantially mitigated the Aβ‐induced synaptic dysfunction (Figure 4A–D), implying that ramalin exerts a protective effect against Aβ toxicity.

Additionally, we examined the size of the total recycling synaptic vesicle pool using prolonged stimulation with 2,000 action potentials (APs) in the presence of bafilomycin, which blocks vesicle reacidification. In neurons treated with Aβ oligomers, the size of the recycling vesicle pool was reduced to ∼38% of normal levels. Notably, ramalin treatment restored the recycling vesicle pool to levels comparable to untreated controls (Figure 4E, F). These data indicate that ramalin preserves both the functional and structural integrity of synaptic vesicles under AD‐related pathological conditions, further highlighting its potential as a therapeutic agent for synaptic protection in AD.

### 2.6 Ramalin Ameliorates the AD Pathology in APP/PS1 Mice

**FIGURE 5:**
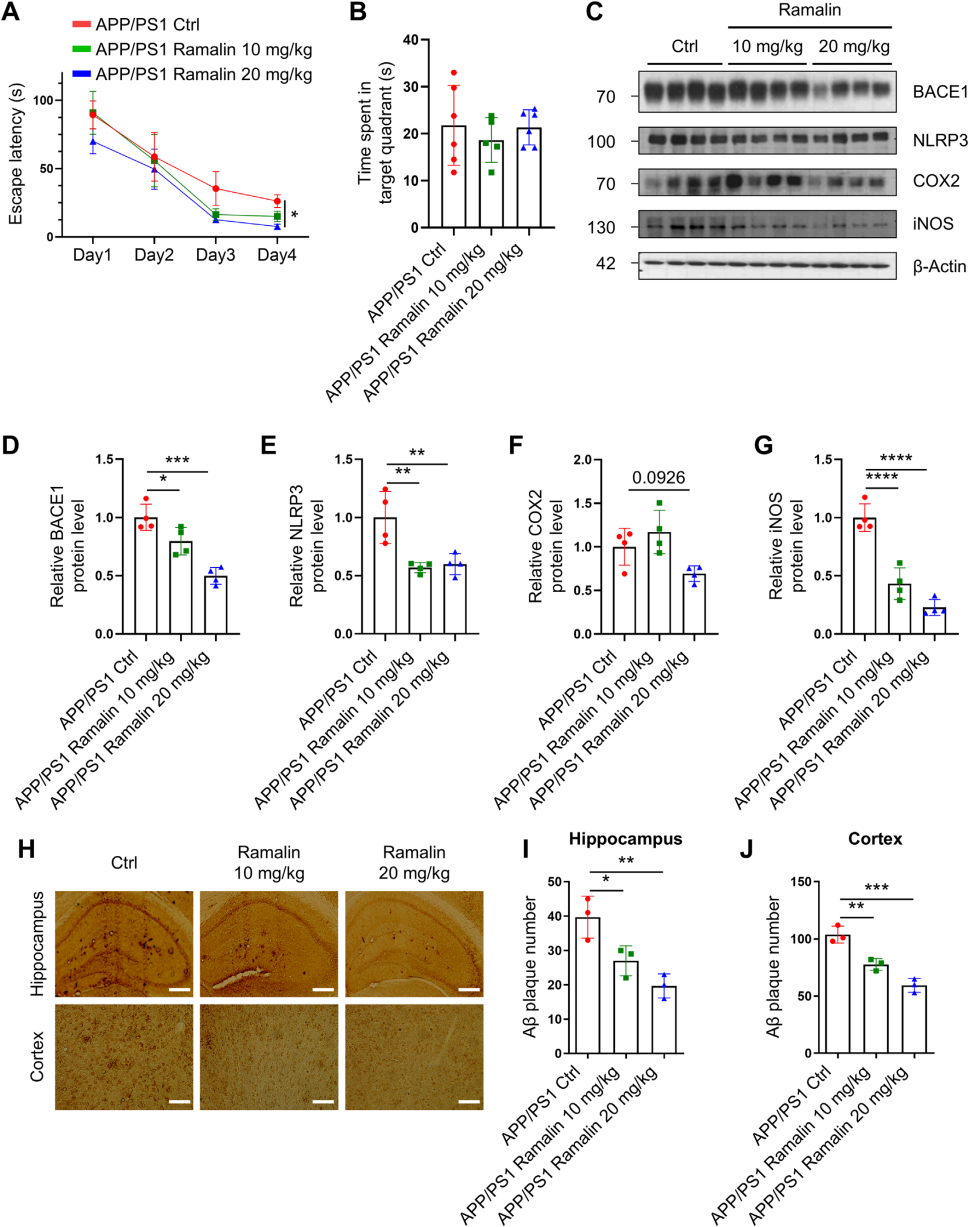
Ramalin restores AD pathology in APP/PS1 mice. APP/PS1 mice were fed with control (APP/PS1 Ctrl) or 10 mg/kg of ramalin (APP/PS1 ramalin 10 mg/kg) or 20 mg/kg of ramalin (APP/PS1 ramalin 20 mg/kg) for 2 months followed by MWM test. (A) Escape latency of mice in MWM test conducted for four consecutive training session (APP/PS1 Ctrl; *n *= 6, APP/PS1 ramalin 10 mg/kg; *n *= 5, APP/PS1 ramalin 20 mg/kg; *n *= 6). (B) Time spent in target quadrant on the probe day (APP/PS1 Ctrl; *n *= 6, APP/PS1 ramalin 10 mg/kg; *n *= 5, APP/PS1 ramalin 20 mg/kg; *n *= 6). (C) Western blot analysis of BACE1, NLRP3, COX2, and iNOS protein levels in brain homogenates of APP/PS1 (*n *= 4 per group). (D–G), Quantification of BACE1 protein levels in (D), NLRP3 protein levels in (E), COX2 protein levels in (F) and iNOS protein levels in (G) from (C) (*n *= 4 per group). The expression levels of each protein were normalized to the expression level of β‐actin. (H) Representative DAB staining images of Aβ plaques in the brains of APP/PS1 mice (scale bar = 25 µm). (I and J) Quantification of Aβ plaque numbers in the hippocampus (I) and cortex (J) shown in (H) (*n *= 3 per group). Data are shown as mean ± SEM in (A), and as mean ± SD in (B), (D), (E), (F), (G), (I), and (J). Data were analyzed using two‐way ANOVA followed by Dunnett's test in (A) and one‐way ANOVA followed by Dunnett's test in (B), (D), (E), (F), (G), (I), and (J). *p < 0.05; **p < 0.01; ***p < 0.001; ****p < 0.0001.

To assess the in vivo efficacy of ramalin, we administered ramalin orally to 7‐month‐old APP/PS1 mice for 2 months and conducted behavioral and biochemical analyses. In the Morris water maze (MWM), ramalin‐treated mice demonstrated significantly improved spatial memory and learning performance compared with control‐treated mice (Figure 5A, B), indicating cognitive benefits from ramalin treatment. Importantly, body and organ weights did not differ significantly among groups, suggesting that ramalin is non‐toxic at the administered doses (Figure S6A, B).

Postbehavioral analysis of brain tissue revealed that ramalin treatment resulted in a significant reduction in BACE1 protein levels, with approximately a 50% decreased compared with the control‐treated mice (Figure 5C, D). Furthermore, ramalin significantly attenuated neuroinflammatory markers, including NLRP3 and iNOS (Figure 5C, E, G). Although ramalin did not significantly reduce COX2 levels, its downregulation of iNOS was dose‐dependent, indicating selective modulation of inflammatory pathways (Figure 5C, F, G).

In addition to its anti‐inflammatory effects, ramalin substantially reduced Aβ plaque burden, as shown by immunohistochemical analysis (Figure 5H–J). These findings suggest that ramalin not only improves cognitive function but also ameliorates key pathological features of AD, including Aβ accumulation and neuroinflammation, in APP/PS1 mice.

### 2.7 Ramalin Reverses Cognitive Impairment by Reducing BACE1 and Phosphorylated Tau in 3xTg‐AD Mice

**FIGURE 6:**
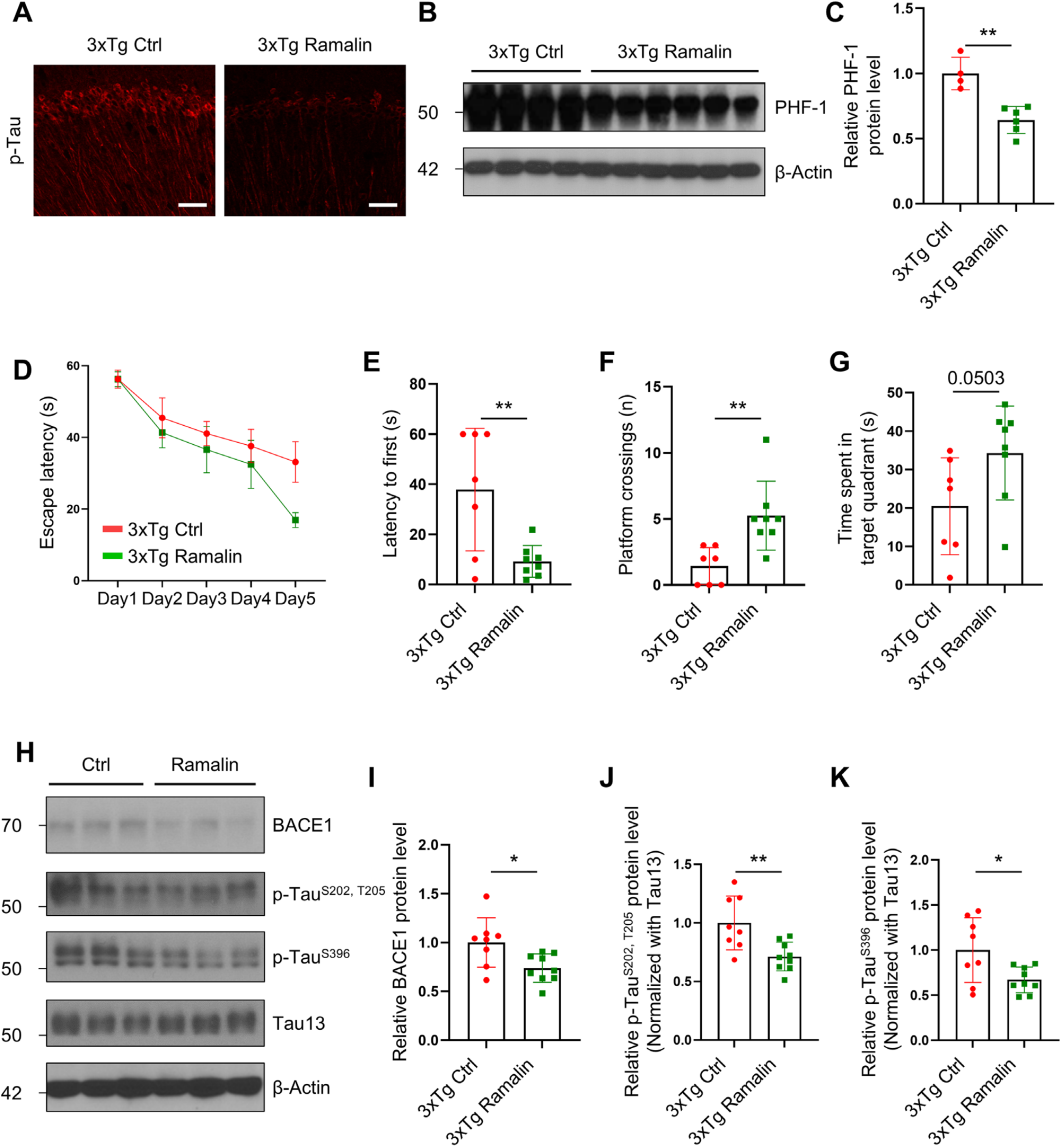
Ramalin mitigates cognitive dysfunction by modulating BACE1 and phosphorylated tau in 3xTg‐AD mice. Young‐aged 3xTg‐AD mice were fed with control (3xTg Ctrl) or 20 mg/kg of ramalin (3xTg Ramalin) for 1 month and old‐aged 3xTg‐AD mice were orally administered with control (3xTg Ctrl) or 20 mg/kg or ramalin (3xTg Ramalin) for 2 months. (A) Immunohistochemistry analysis of p‐Tau in the brains of young‐aged 3xTg‐AD group (scale bar = 25 µm). (B) Western blot analysis of PHF‐1 protein levels in the brain homogenates of young‐aged 3xTg‐AD group. (C) Quantification of PHF‐1 protein levels in (B) (3xTg Ctrl; *n *= 4, 3xTg ramalin; *n *= 6). The expression level of PHF‐1 was normalized to the expression level of β‐actin. (D) Escape latency of old‐aged 3xTg‐AD group in MWM test conducted for five consecutive training session (3xTg Ctrl; *n *= 7, 3xTg ramalin; *n *= 8). (E) Latency to first visit to platform of old‐aged 3xTg‐AD group on probe day (3xTg Ctrl; *n *= 7, 3xTg ramalin; *n *= 8). (F) The number of platform crossings on the probe day in old‐aged 3xTg‐AD mice (3xTg Ctrl; *n *= 7, 3xTg ramalin; *n *= 8). (G) Time spent in the target quadrant on the probe day in old‐aged 3xTg‐AD mice (3xTg Ctrl; *n *= 7, 3xTg ramalin; *n *= 8). (H) Representative western blot analysis of BACE1, p‐Tau^S202, T205^, p‐Tau^S396^ and Tau13 protein levels in the hippocampus of old‐aged 3xTg‐AD mice. (I–K) Quantification of BACE1 in (I), p‐Tau^S202, T205^ (AT8) in (J), and p‐Tau^S396^ in (K) from the hippocampus of old‐aged 3xTg‐AD mice (3xTg Ctrl; *n *= 8, 3xTg ramalin; *n *= 9). The expression level of BACE1 was normalized to the expression level of β‐actin, and the expression level of p‐Tau^S202, T205^, and p‐Tau^S396^ was normalized to the expression level of Tau13. Data are shown as mean ± SD in (C), (E), (F), (G), (I), (J), and (K) and as mean ± SEM in (D). Data were analyzed using two‐way ANOVA followed by Dunnett's test in (D) and unpaired two‐tailed *t* test in (C), (E), (F), (G), (I), (J), and (K). *p < 0.05; **p < 0.01.

To extend our findings on ramalin's efficacy in AD, we evaluated its effects on tau pathology using the 3xTg‐AD mouse model, which exhibits both Aβ and tau pathology. We administered ramalin (20 mg/kg) orally to 7‐month‐old (young‐aged) and 16‐month‐old (old‐aged) 3xTg‐AD mice and conducted behavioral and biochemical analyses. In young‐aged mice, ramalin significantly reduced phosphorylated tau in the CA1 region of the hippocampus (Figure 6A) and lowered PHF‐1, which detects tau phosphorylated at Ser396 and Ser404, levels in brain homogenates (Figure 6B, C). In old‐aged mice, ramalin improved learning performance during the MWM training session and mitigated spatial memory deficits, as shown by probe trials (Figure 6D–G).

Biochemical analysis of brain tissue from old‐aged 3xTg‐AD mice revealed that ramalin reduced both BACE1 protein and phosphorylated tau levels in the hippocampus (Figure 6H–K). These results suggest that ramalin may exert its neuroprotective effects by targeting both amyloidogenic and tauopathic processes, thereby improving cognitive outcomes in AD models.

### 2.8 Transcriptomic Changes Induced by Ramalin Treatment

**FIGURE 7:**
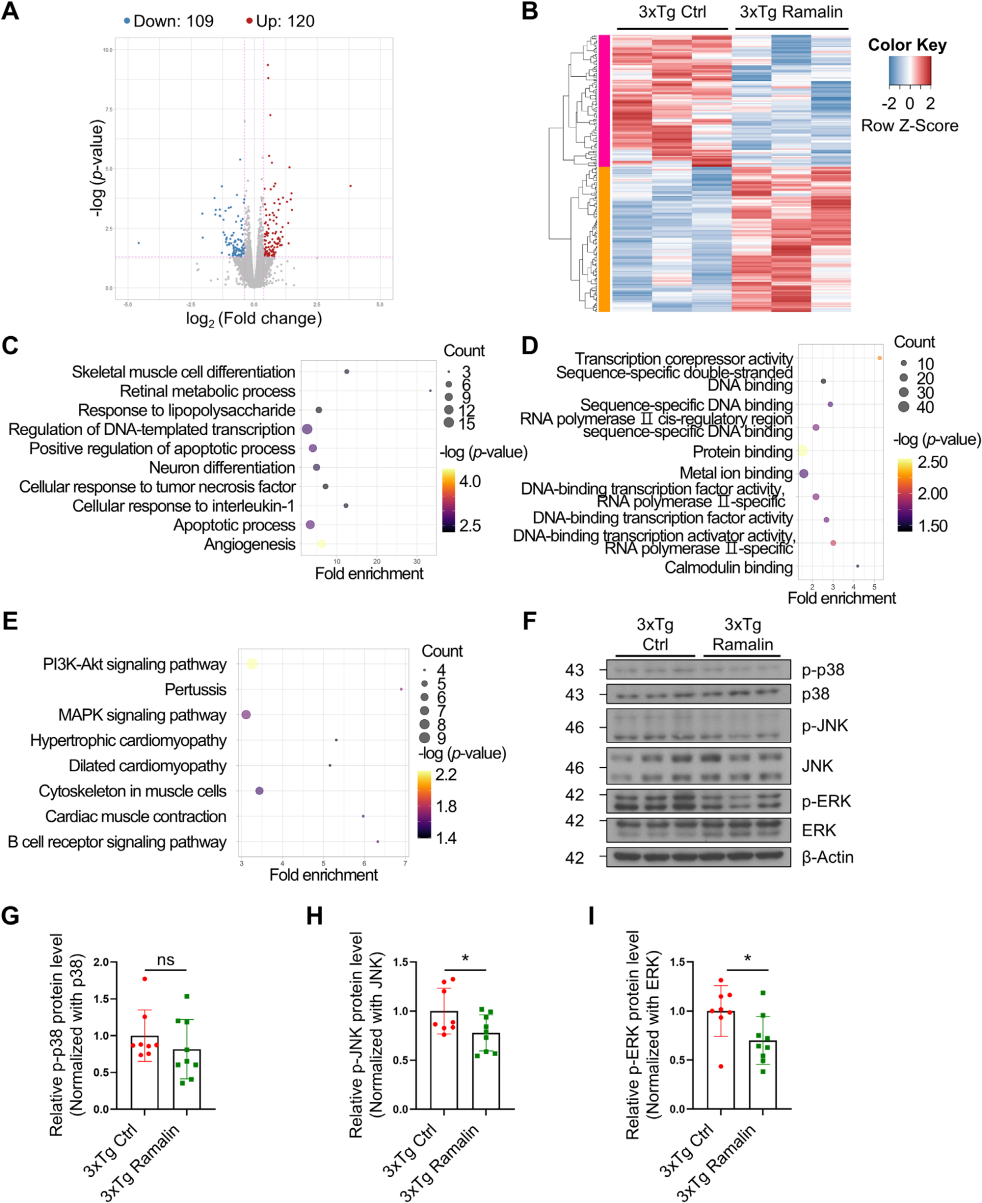
Transcriptomic analysis of 3xTg‐AD reveals ramalin modulates MAPK signaling pathway. RNA‐sequencing analysis revealed that 229 differentially expressed genes (DEGs) displayed as a volcano plot in (A) and as a heatmap in (B). Fold change is same or higher than 1.3 and *p* value is lower than 0.05 is regarded as differentially expressed. (C) Top 10 enriched terms in biological process of GO analysis using DEGs. (D) Top 10 enriched terms in molecular function of GO analysis using DEGs. (E) Top 8 enriched terms in KEGG pathway analysis using DEGs. (F) Representative western blot analysis of p‐p38, p38, p‐JNK, JNK, p‐ERK, and ERK protein levels in the hippocampus of old‐aged 3xTg‐AD mice. (G–I) Quantification of p‐p38/p38 in (G), p‐JNK/JNK in (H) and p‐ERK/ERK in (I) from the hippocampus of old‐aged 3xTg‐AD mice (3xTg Ctrl; *n *= 8, 3xTg ramalin; *n *= 9). Data are shown as mean ± SD in (G–I). Data were analyzed using unpaired two‐tailed *t* test in (G–I). ns; *p* > 0.05; *p < 0.05.

To gain insights into the molecular mechanisms underlying therapeutic effects of ramalin, we performed transcriptomic analysis using RNA sequencing from the cortices of ramalin‐treated 3xTg‐AD mice. We identified 229 differentially expressed genes (DEGs), with 120 upregulated and 109 downregulated in response to ramalin treatment (Figure 7A, B). Gene Ontology (GO) analysis revealed significant enrichment in biological processes such as “Cellular response to tumor necrosis factor, ” “Response to lipopolysaccharide, ” and “Apoptotic process” (Figure 7C) [52, 53]. Among molecular function terms, genes associated with “Calmodulin binding” and “DNA binding” were significantly enriched (Figure 7D). Functional enrichment analysis based on the Kyoto Encyclopedia of Genes and Genomes (KEGG) database revealed that genes were associated with the “MAPK signaling pathway, ” a critical regulator of cellular stress responses and inflammation (Figure 7E) [54].

Although ramalin attenuated the NLRP3 inflammasome activation in APP/PS1 mice, transcriptomic analyses of 3xTg‐AD cortices revealed no significant alterations in disease‐associated microglia (DAM) markers. Expression patterns of these genes are visualized, but no statistically significant differences were detected (Figure S7A). Furthermore, real‐time quantitative PCR (RT‐qPCR) analysis of cortical tissue showed no significant differences in the mRNA expression levels *Gfap*, *Iba1*, *Il1a*, and *Il6* between vehicle‐ and ramalin‐treated groups (Figure S7B–E). These findings indicate that ramalin's anti‐inflammatory effects were not accompanied by broad transcriptional shifts in DAM‐associated genes or classical glial/inflammatory markers.

### 2.9 Ramalin Regulates the MAPK Signaling Pathway

The MAPK signaling pathway has been strongly implicated in AD, particularly in the phosphorylation of tau protein by kinases such as JNK, p38 MAPK, and ERK [35]. We examined phosphorylated MAPK in the hippocampi of old‐aged 3xTg‐AD mice treated with ramalin. Ramalin significantly decreased levels of phosphorylated JNK and phosphorylated ERK (Figure 7F–I), indicating reduced activation of these stress‐associated kinases. Although the reduction in phosphorylated p38 MAPK was 19%, this decrease did not reach statistical significance (Figure 7F, G). These findings were consistent in young‐aged 3xTg‐AD mice, where ramalin significantly reduced phosphorylated JNK and ERK (Figure S8A, D, E), and in APP/PS1 mice, where p‐ERK levels were also significantly reduced (Figure S9A, D).

From these results, it seems that ramalin exerts a neuroprotective effect in AD, most likely by affecting MAPK signaling and thereby decreasing tau phosphorylation.

## 3 Discussion

For decades, Aβ has been the primary focus of therapeutic strategies in AD. The aggregation of Aβ monomers into toxic oligomers disrupts neuronal structures such as dendrites and synapses, contributing to AD pathogenesis. APP is cleaved by BACE1, which controls the rate of Aβ generation. In AD brains, both the expression and enzymatic activity of BACE1 are markedly higher than in non‐demented controls, making this enzyme an important focus in drug discovery efforts [55]. Recent study has shown that reducing BACE1 expression through epigenome editing can ameliorate cognitive dysfunction and reduce amyloid plaques in AD mouse models [56]. In our study, we demonstrated that ramalin effectively downregulates BACE1 expression, offering protective effects against oxidative stress and Aβ oligomers in both cellular models and AD animal models. However, our data indicate that ramalin does not alter BACE1 at the transcription or translational levels, as evidenced by its lack of effect on BACE1 mRNA expression, promoter activity, or UTRs. Thus, our findings indicate that ramalin decreases BACE1 protein levels through posttranslational mechanisms, independent of conventional transcriptional or translational control.

Neuroinflammation has emerged as a key contributor to AD pathogenesis, with activation of the NLRP3 inflammasome playing a central role. Inhibiting NLRP3 has been shown to alleviate AD‐related pathology [20, 23]. Our findings reveal that ramalin exerts significant anti‐inflammatory effects, particularly by downregulation of NLRP3 at both the mRNA and protein levels. This reduction in NLRP3 activation led to decreased levels of downstream proteins, including caspase‐1 and IL‐1β. Additionally, ramalin reduced iNOS expression and its byproduct NO, both of which are associated with neurodegenerative processes in AD. These effects were observed in both BV‐2 microglial cells and in brain homogenates from APP/PS1 mice, further supporting the potential of ramalin as an anti‐inflammatory agent in AD.

Importantly, our study demonstrated that ramalin can prevent Aβ oligomer‐induced synaptic transmission impairment, a major pathological event that contributes to cognitive deficits in AD. Using a pHluorin‐based assay, we found that ramalin not only restored synaptic transmission but also preserved the pool of vesicles available for release in hippocampal neurons exposed to Aβ oligomers. This preservation of synaptic function suggests that ramalin may help maintain cognitive abilities by preventing the progression of synaptic deficits, a hallmark of AD.

The therapeutic potential of ramalin was further evaluated in vivo using APP/PS1 and 3xTg‐AD mouse models. The APP/PS1 model, recapitulating amyloid pathology, revealed that ramalin treatment improved spatial learning and memory performance, as demonstrated by MWM testing. These cognitive improvements were accompanied by a significant reduction in BACE1 expression and a corresponding decrease in Aβ plaque accumulation. Similarly, in the 3xTg‐AD model, which harbors mutations in *APP*, *Psen1*, and *MAPT*, ramalin improved cognitive function, significantly reduced hippocampal BACE1 levels, and decreased phosphorylation of tau at residues S202, T205, S396, and S404. These findings highlight the potential of ramalin to target both amyloidogenic and tauopathic processes in AD.

Transcriptome analysis of ramalin‐treated 3xTg‐AD mice identified 229 DEGs, with GO and KEGG pathway analyses revealing significant enrichment in the MAPK signaling pathway. This pathway has been implicated in tau hyperphosphorylation and AD progression, with kinases such as JNK, ERK, and p38 MAPK playing key roles in phosphorylating tau at disease‐relevant sites [36, 37, 38]. Notably, ramalin treatment significantly reduced the phosphorylated forms of JNK, ERK, and, to a lesser extent, p38 MAPK in both 3xTg‐AD and APP/PS1 mouse brains. Previous studies have demonstrated that JNK activation is a critical driver of tau hyperphosphorylation at S202/T205, and inhibiting JNK reduces tau phosphorylation and ameliorates AD pathology [36]. Our findings suggest that ramalin may exert its therapeutic effects, in part, by mitigating MAPK pathway activation and thus reducing tau phosphorylation.

While ramalin demonstrated clear benefits in terms of cognitive function and reductions in key AD‐related pathologies—including Aβ plaques, phosphorylated tau, and neuroinflammation—the precise molecular mechanisms underlying its effects require further elucidation. To identify potential molecular targets of ramalin, we performed *GPScreen* in *S*. *pombe*, which revealed several candidates including HDACs. These enzymes remove acetyl groups from lysine residues on histones as well as other cellular proteins. HDACs are highly conserved proteins and play important roles in gene transcription and various cellular processes. Aberrant HDAC activity has been implicated in diverse diseases, including tumors, ocular diseases and AD [26, 30, 57, 58]. Particularly, HDAC6 has been implicated in AD pathology, with elevated HDAC6 levels observed in AD brains [28]. HDAC6 interacts with tau and modulates its phosphorylation, and genetic deletion or pharmacological blockade of HDAC6 decreases Aβ and tau pathology and consequently improving cognitive performance in AD animal models [30, 31]. Moreover, HDAC6 is involved in NLRP3 inflammasome pathway [32]. HDAC6 plays a critical role in the assembly and activation of NLRP3 inflammasome [32]. Genetic ablation of *Hdac6* and pharmacological inhibition of HDAC6 hindered NLRP3 inflammasome activation and IL‐1β release [32, 33]. Our results also suggest that HDAC6 influences BACE1 protein expression; however, whether this occurs through direct interaction or indirect pathways remain unclear. Future investigations will be necessary to clarify the precise mechanism. Consistent with the *GPScreen* findings, our HDAC activity assay confirmed that ramalin selectively inhibits HDAC6 over other isoforms. This selectivity likely contributes to the observed reductions in BACE1, tau phosphorylation, NLRP3 activation, and overall AD pathology, positioning HDAC6 as a key mediator of ramalin's neuroprotective effects.

While our findings provide compelling evidence for the therapeutic potential of ramalin in AD, several limitations should be acknowledged. First, the pharmacokinetic profile and blood–brain barrier permeability of ramalin were not systematically investigated, leaving uncertainties regarding its bioavailability in the central nervous system. Second, long‐term safety and toxicity remain to be established, which are critical for assessing its feasibility as a therapeutic agent. Finally, given that ramalin targets multiple pathways, the possibility of off‐target effects cannot be excluded and should be carefully evaluated in future studies. Addressing these pharmacological and translational issues will be crucial to clarify the therapeutic applicability of ramalin in AD.

Overall, our findings indicate that ramalin confers therapeutic benefits in AD models through coordinated modulation of BACE1, neuroinflammation, and tau abnormalities. These actions collectively ameliorate cognitive impairments associated with AD. Given these promising results, ramalin represents a potential therapeutic agent for the treatment of AD, although further studies are needed to fully elucidate its mechanisms of action and to assess its efficacy in clinical settings.

## 4 Conclusion

This study underscores the diverse pharmacological effects of ramalin in mitigating multiple pathological aspects of AD. Ramalin decreases BACE1 protein levels via selectively inhibiting HDAC6. Ramalin suppresses NLRP3 inflammasome pathway and reduces inflammatory markers like iNOS/NO and IL‐1β. Additionally, ramalin restores synaptic transmission defects induced by oAβ and downregulates tau phosphorylation in 3xTg‐AD. Furthermore, it ameliorates cognitive dysfunction in AD mouse models, both in APP/PS1 and 3xTg‐AD. Transcriptomic analysis reveals that ramalin modulates MAPK pathway, which plays a crucial role in regulating tau phosphorylation. Collectively, these results position ramalin as a promising candidate for AD therapy.

## 5 Materials and Methods

### 5.1 Animals

APP/PS1 (B6.Cg‐Tg(APPswe, PSEN1dE9)85Dbo/Mmjax) mice and 3xTg‐AD (B6.Cg‐Tg(APPSwe, tauP301L)1Lfa *Psen1^1Mpm^*/2J) mice were used in this study. All mice were housed under a 12‐h light/dark schedule, with food and water available ad libitum. At 7 months of age, APP/PS1 mice were treated orally with 10 or 20 mg/kg of ramalin once daily for a month. At 7 months of age and 16 months of age, ramalin was administered orally to 3xTg‐AD mice at a daily dose of 20 mg/kg for duration of one and two months, respectively. Following treatment, the mice underwent the MWM. Female APP/PS1 mice and both male and female 3xTg‐AD mice were used in this study. All animal experiments were conducted in accordance with protocols approved by the Institutional Animal Care and Use Committee of Sungkyunkwan University (SKKUIACUC2021‐06‐44‐1).

### 5.2 MWM Test

The MWM test was used to assess the learning ability and spatial memory of the animals. This test was carried out following previously reported procedures with minor modifications [59]. The test consisted of training session conducted for 4 consecutive days (APP/PS1) or 5 consecutive days (3xTg‐AD) and a 1‐day probe session. A circular pool, 150 cm in diameter, was used for the experiment. Before tests, the pool was filled so that the water level stood about 1 cm above the platform, and harmless white dye was mixed to make the platform invisible to the mice. The temperature of water was set to 22–24°C. The pool area was divided into four quadrants, with one quadrant containing the hidden platform. Four visual cues were placed on the wall of pool throughout the test. During the training session, mice were placed in the pool from different starting points two times per day (APP/PS1) or three times per day (3xTg‐AD). The latency to reach the platform and swimming trajectories were monitored by a video tracking system. Trials were limited to 120 s (APP/PS1) or 60 s (3xTg‐AD). When the mouse reached the platform within the trial and remained there for 5 s, it was placed back into its home cage. Mice that failed to reach the platform within the trial were gently guided to the platform and allowed to explore the environment for 10 s. The probe session was conducted 24 h after the last training session. In the probe session, platform was removed, and the mice were placed on the opposite side of where the platform had been. Mice were allowed to swim for 120 s (APP/PS1) or 60 s (3xTg‐AD). Each trial was analyzed using Ethovision software (Ethovision).

### 5.3 Brain Tissue Collection and Processing

Following MWM, mice were placed under anesthesia with a Zoletil (Virbac)–Rompun (Bayer) mixture and perfused with PBS as previously described [60]. Brain tissues were extracted and bisected. One was immersed in 4% paraformaldehyde for fixation. The cortex and hippocampus were gently isolated from the opposite side, snap‐frozen in liquid nitrogen and kept in deep freezer for later biochemical analysis.

### 5.4 Plasmid Construction

The GFP^u^ vector was generously gifted from Dr Yong Keun Jung (Seoul National University, Republic of Korea) and 5′ UTR–BACE1–3′ UTR expressing construct was kindly provided from Dr Christian Haass (DZNE, Germany). BACE1 and GFP series constructs used in this study were cloned into pcDNA3.1 using appropriate combinations of *Hin*dIII, *Not*I, *Eco*RI, and *Xho*I restriction sites.

### 5.5 Cell Culture

Human neuroblastoma cell line, SH‐SY5Y, human monocytic cell line, THP‐1, human embryonic kidney cell line, HEK293T, mouse embryonic fibroblast, MEF, and mouse microglial cell line, BV‐2 were maintained in either DMEM (Hyclone) or RPMI1640 (Corning) containing 10% fetal bovine serum (FBS; Gibco) and 1% penicillin–streptomycin solution (P/S; Capricorn), and incubated at 37°C under 5% CO_2_ (v/v) in a humidified chamber. Heat‐inactivated FBS was used for the MEF and BV‐2.

### 5.6 Primary Neuron Culture

Primary neurons were cultured using embryos of Sprague–Dawley rat at day 17 as previously described with some modifications [61]. Briefly, brains were extracted, and the meninges were removed. The tissues were incubated with trypsin for 15 min at 37°C, gently homogenized by pipetting, and then passed through a 100‐µm cell strainer. Primary neurons were cultured on poly‐d‐lysine (Sigma)‐coated plates in DMEM containing 10% FBS and 1% P/S. On the next day, the media was replaced with neurobasal media (Gibco) supplemented with 1% P/S and B‐27 supplement (Gibco). The media was half‐changed every other day for 10 days. After 10 days, the primary neurons were treated with ramalin in dose‐dependent manner. For the optical imaging experiments, the hippocampus was dissected from Sprague–Dawley rat pups (P0–P3) and cultured on poly‐ornithine‐coated glass coverslips. Neuronal transfections were conducted on day 7 after plating and subsequently maintained in culture for an additional 14–21 days. All results were obtained from three or more independent primary neuron preparations.

### 5.7 Primary Microglia Culture

Primary microglia were cultured using postnatal day 1 C57BL/6 mice [62]. In brief, brains were extracted, and the meninges were removed. The brains were then incubated with 0.25% trypsin–EDTA for 15 min at 37°C and subsequently washed four times with DMEM/F12 (Gibco) containing 10% FBS, 1% P/S, 1% l‐glutamine, 1% sodium pyruvate, and 1% nonessential amino acids. The brains were homogenized by pipetting and subsequently passed through a 70 µm cell strainer. The filtered cells were plated in T‐75 flasks, with media changes occurring after 6 days. On the 16th day after plating, primary microglia were isolated from the T‐75 flasks using the EasySep Mouse CD11b Positive Selection Kit II (Stemcell Technologies). Media was removed from the flasks and 3 mL of 0.25% trypsin–EDTA was added to each flask and incubated at 37°C for 5 min. Media was then added to the flasks and the cells were resuspended and filtered through a 70 µm cell strainer. The filtrate was centrifuged at 120 g for 5 min and the supernatant was removed. The pellet was resuspended in 1 mL of DPBS (Sigma) containing 2% FBS and 1 mM of EDTA and transferred to the 5 mL polystyrene tube. A total of 50 µL of rat serum was added to the suspension and incubated for 5 min at room temperature. The tube was incubated in the magnet for 3 min at room temperature. While the tube was placed on a magnetic stand, the DPBS was removed, and the washing procedure was repeated. The cell pellets were resuspended with 3 mL of DMEM/F12 media and seeded in poly‐d‐lysine‐coated plates. Primary microglia were exposed to 20 µM of ramalin for 1 day. Then, 0.2 µg/mL of LPS (Sigma) was added for 6 h, followed by the addition of 20 µM of nigericin (AdipoGen Life Sciences) for 30 min.

### 5.8 Optical Imaging with Ramalin Treatment

For optical imaging, primary mouse hippocampal neurons were cultured and transfected with vG–pH through calcium phosphate‐mediated precipitation, as previously reported [63, 64]. vG–pH was mixed with 2× HeBS solution (273 mM of NaCl, 9.5 mM of KCl, 1.4 mM of Na_2_HPO_4_·7H_2_O, 15 mM of d‐glucose, 42 mM of HEPES, pH adjusted to 7.10) supplemented with 2 mM of Ca^2+^, and the solution was administered to neurons on DIV8. Neurons at DIV14‐21 were subjected to live‐cell imaging following exposure to 1 µM of Aβ only or 1 µM of Aβ with 5 µM of ramalin for 16 h. For imaging, neuron‐bearing coverslips were transferred to a perfusion chamber under continuous laminar flow and imaged using a custom‐designed epifluorescence microscope (Zeiss Observer) equipped with a 488 nm OBIS laser (Coherent) for excitation. Images were captured using an Andor iXon Ultra 897 EMCCD camera. The laser output was shuttered in synchrony with the EMCCD through the TTL on/off signal during image acquisition. Fluorescence signals were excited and collected using a Zeiss 40× Fluar objective (1.3 NA) in combination with a 498 nm dichroic mirror and 500–550 nm emission filters (Chroma). To elicit APs, 1‐ms current pulse were delivered through platinum‐iridium electrodes connected to an isolated pulse stimulator (World Precision Instruments). During recordings, neurons were continuously superfused with Tyrode's solution containing 119 nM of NaCl, 2.5 mM of KCl, 2 mM of CaCl_2_, 2 mM of MgCl2, 25 mM of HEPES, 30 mM of glucose, 10 µM of 6‐cyano‐7‐nitroquinoxaline‐2, 3‐dione, and 50 µM of d, l‐2‐amino‐5‐phosphonovaleric acid (AP5), and titrated to pH 7.4. Experiments were conducted at 30°C, and images were captured at 2 Hz with an exposure time of 50 ms.

### 5.9 Image Analysis

Image analyses were conducted with ImageJ utilizing Time Series plugin. Elliptical ROIs (10‐pixel diameter) representing synaptic boutons were used for fluorescence measurements. Subsequently, fluorescence intensity at synapses was quantified. Analysis of fluorescence signal was performed using Origin Pro.

### 5.10 Aβ Oligomer Preparation

Aβ oligomer (oAβ) was prepared as previously described [59]. Briefly, Aβ_1–42_ (Anygen) was initially dissolved in 1, 1, 1, 3, 3, 3‐hexafluoro‐2‐propanol (Sigma). The dissolved Aβ_1–42_ was then aliquoted and lyophilized in a fume hood. Lyophilized Aβ_1–42_ was stored in deep freezer until experiments. For experiments, lyophilized Aβ_1–42_ was solubilized in DMSO (Sigma) and subsequently diluted to 100 µM in DMEM/F‐12 (Gibco). The diluted Aβ_1–42_ was incubated at 4°C for 1 day. SH‐SY5Y cells were then incubated with 2 µM of oAβ with or without 5 µM of ramalin for 24 h.

### 5.11 Western Blot Analysis

Western blot analysis was performed as previously described [65]. For in vitro experiments, cells were lysed in T‐PER (Thermo Scientific) containing a protease/phosphatase inhibitor cocktail (Thermo Scientific) and kept on ice for 10 min. Cell debris was removed by centrifugation at 15,000 g at 4°C for 10 min, and the resulting supernatant was collected for subsequent western blot analysis. For in vivo samples, mouse brain tissues were homogenized in RIPA buffer (Millipore) containing protease/phosphatase inhibitor cocktail. The homogenates were then kept on ice for 30 min and centrifuged 15,000 g at 4°C for 30 min. The supernatant was obtained for western blot analysis. Protein concentration was determined using a BCA assay kit (Thermo Scientific). Lysates were then mixed with LDS Sample Buffer (Invitrogen) and 5% 2‐mercaptoethanol (Sigma), heated to 95°C for 5 min. Equal amounts of protein (8–12 µg) were loaded on SDS‐polyacrylamide gels for separation and electrophoresed. After SDS‐PAGE, the proteins were transferred to polyvinylidene fluoride (PVDF) membranes. PVDF membranes were then incubated in a blocking buffer containing 5% nonfat dry milk at room temperature for 1 h. Following blocking, the PVDF membranes were incubated at 4°C for overnight with primary antibodies against Ac‐tubulin (Sigma), BACE1 (Cell Signaling Technology), β‐actin (Invitrogen), caspase‐1 (AdipoGen Life Sciences), COX2 (Abcam), ERK (Cell Signaling Technology), GFP (Santa Cruz Biotechnology), HDAC6 (Cell Signaling Technology), IL‐1β (R&D Systems), iNOS (Santa Cruz Biotechnology), JNK (Cell Signaling Technology, Epitomics), LC3 (Novus Biologicals), NLRP3 (Cell Signaling Technology, ProSci), p‐ERK (Cell Signaling Technology), p‐JNK (Cell Signaling Technology), p‐p38 (Cell Signaling Technology), p‐Tau^S202, T205^ (AT8; Invitrogen), p38 (Cell Signaling Technology), PHF‐1 (In‐house), Tau13 (BioLegend), and tubulin (Cell Signaling Technology). After incubation with the primary antibodies, the membranes were rinsed three to four times in tris‐buffered saline containing 1% (v/v) Tween‐20 (TBS‐T) and exposed to HRP‐linked secondary antibodies against mouse or rabbit IgG (Millipore) for 1 h at room temperature. Protein bands were visualized using ECL solution (Cytiva). Protein signal intensity was quantified using ImageJ 1.54f (NIH).

### 5.12 RNA Extraction and RT‐qPCR

Total RNA was purified from cultured cells or mouse brain samples using RNAiso Plus (Takara) according to the supplier's instructions. Reverse transcription was performed using the PrimeScript RT Reagent Kit equipped with a genomic DNA removal step (Takara), ensuring equal RNA input across samples. The resulting cDNA was subsequently diluted ten‐fold in RNase free water prior to RT‐qPCR. RT‐PCR was performed using TB Green Premix Ex Taq II (Takara) and a CFX Connect (Bio‐rad). Gene expression levels were normalized to reference genes (*GAPDH* or *Hprt*). The sequences of primers used for RT‐qPCR were as follows: human *BACE1* forward 5′‐gtcatccacgggcactgt‐3′, human *BACE1* reverse 5′‐ccgtcctgaactcatcgtg‐3′, human *GAPDH* forward 5′‐catcaatggaaatcccatca‐3′, human *GAPDH* reverse 5′‐gactccacgacgtactcagc‐3′, mouse *Nlrp3* forward 5′‐tgctcttcactgctatcaagccct‐3′, mouse *Nlrp3* reverse 5′‐acaagcctttgctccagaccctat‐3′, mouse *Hprt* forward 5′‐cctcctcagaccgcttttt‐3′, and mouse *Hprt* reverse 5′‐aacctggttcatcatcgctaa‐3′.

### 5.13 Immunohistochemistry

The hemispheres stored in 4% paraformaldehyde were cryopreserved in 30% sucrose for 48 h at 4°C. Samples were frozen after embedding in OCT and cut into 45 µm‐thick slices. DAB staining was conducted following the manufacturer's protocol. To investigate Aβ deposition, we immunostained tissue samples with a primary antibody against Aβ (Covance). The number of Aβ plaques was quantified with ImageJ software (NIH).

### 5.14 BACE1 Activity Assay

The BACE1 activity assay was conducted using the β‐Secretase (BACE1) Activity Detection Kit (Sigma) following the manufacturer's protocols. A total of 50 µM of BACE1 substrate and 0.3 units/µL of BACE1 enzyme were diluted in the same buffer. Ramalin was serially diluted in the buffer. BACE1 substrate, BACE1 enzyme, ramalin and buffer were added to the wells to a final volume of 100 µL. Initially, the fluorescence value (ex/em 320 nm/405 nm) was recorded, and after 2 h of incubation at 37°C, the fluorescence value was recorded again.

### 5.15 BACE1 Promoter Luciferase Assay

The BACE1 promoter luciferase assay was conducted as described previously [16]. Briefly, HEK293T cells were transfected with either the WT or deletion constructs of human *BACE1* promoter reporter plasmids and the Renilla luciferase plasmid using polyethyleneimine. One day after transfection, cells were exposed to either vehicle or 10 µM of ramalin for 24 h. Then, cells were lysed with passive lysis buffer (Promega) and the luminescence of firefly and Renilla were measured using a luminometer (Berthold Detection Systems) with the Dual‐Luciferase Reporter Assay System (Promega). Relative luciferase activity was calculated by dividing firefly luminescence by Renilla luminescence values.

### 5.16 NO Measurement

NO concentration in the cultured media was measured using the Griess reaction as previously described with minor modifications [66]. The murine microglial cell line, BV‐2, was incubated with 1 µg/mL of LPS for 1 day with either vehicle or ramalin pretreatment. 50 µL of cultured supernatant were mixed with an equal volume of Griess reagent (0.1% N‐(1‐naphthyl)‐ethylenediamine dihydrochloride and 1% sulfanilamide in 5% phosphoric acid) and incubated for 10 min at room temperature in the dark. Nitrite was quantified from absorbance at 540 nm using standard curves derived from sodium nitrite diluted in the respective culture media.

### 5.17 TNFα Measurement

THP‐1 cells were incubated with phorbol 12‐myristate 13‐acetate to induce differentiation. After differentiation, cells were exposed to 1 µM of ramalin for 1 day, and then 0.1 µg/mL of LPS was added for 4 h. Supernatant were collected, and TNFα concentration was measured using human TNFα ELISA kit (eBioscience) following manufacturer's instructions.

### 5.18 RNA Sequencing Analysis

RNA sequencing analysis was carried out as previously described [67]. Total RNA from mouse cortical tissue (3xTg Ctrl and 3xTg ramalin) was used for mRNA sequencing analysis. mRNA library preparation, transcriptome sequencing, and quality control were performed by Novogene Co., LTD (Hong Kong). Genes showing a fold change of ≥1.3 with a *p* value < 0.05 were considered to be differentially expressed.

### 5.19 GPScreen^TM^

To identify the putative target of ramalin, genome‐wide *GPScreen* was performed by Bioneer Co. (Republic of Korea) [46, 47]. First, the growth‐inhibitory activity (GI_50_) of ramalin was determined using the wild‐type fission yeast *Schizosaccharomyces pombe* (*S.pombe*). Then, potential drug target candidates were initially screened using a library of 1,276 *S. pombe* essential gene deletion mutants. Subsequently, an in‐depth screening was carried out to select the most promising drug target candidates from the 144 initially identified targets.

### 5.20 HDAC Assay

To determine the IC_50_ value of ramalin on HDAC, an HDAC profiling assay was conducted by Reaction Biology Corp (USA). This assay is based on the combination of a fluorogenic substrate and developer. A 10‐step, twofold dilution series from 100 µM was applied to examine ramalin's inhibitory profile on HDAC1‐11, sirtuins (SIRT1, 2, 3, and 5). Substates for HDAC1, 2, 3, 6, 10, SIRT1, 2 and 3 are fluorogenic peptide from p53 residues 379–382 (RHKK(Ac)AMC). Substrates for HDAC4, 5, 7, 9, and 11 are fluorogenic HDAC Class 2a substrate (trifluoroacetyl lysine), and substrate for HDAC8 is a fluorogenic peptide from p53 residues 379–382 (RHK(Ac)K(Ac)AMC). Substrate for SIRT5 is a fluorogenic peptide Ac‐Lys‐succ. IC_50_ values were calculated based on non‐linear regression, where the DMSO‐only control point was assigned to 1.00 × 10^−12^ M for fitting purposes.

### 5.21 Statistical Analysis

Graph preparation and data analyses were conducted using Prism 8 (GraphPad Software). Statistical comparisons among groups were determined with either unpaired *t*‐test or ANOVA, with Tukey's or Dunnett's post hoc evaluation, as appropriate. Data are represented as the mean together with SD or SEM.

## Author Contributions

Y.C. and J.L. analyzed the RNA‐Seq results. Y.C. and J.L. wrote and revised the manuscript. B.Y.C. conducted in vitro experiments and analyzed *GPScreen^TM^* data. J.‐H.Y. conducted in vivo experiments using APP/PS1 mice. S.H. performed synaptic transmission function experiments and wrote the manuscript. S.H.B., J.P., Y.C., and H.K.K. analyzed HDAC inhibitory assay. E.K. analyzed the RNA‐seq results. L.F.P., J.L., Y.J., and J.I. conducted in vitro experiments. J.‐M.H. and T.K.K. synthesized the ramalin. S.H.K. reviewed synaptic transmission function results and the manuscript. J.H.Y. and D.‐G.J. designed and supervised the entire project. D.‐G.J. wrote and edited the manuscript. All authors have read and approved the final manuscript.

## Ethics Statement

All animal experiments were conducted in accordance with protocols approved by the Institutional Animal Care and Use Committee of Sungkyunkwan University (SKKUIACUC2021‐06‐44‐1).

## Conflicts of Interest

The authors declare no conflicts of interest.

## Funding Information

This work was supported by Korea Institute of Marine Science & Technology Promotion (KIMST) grant funded by the Ministry of Oceans and Fisheries, Republic of Korea (RS‐2021‐KS211513), and by the Basic Science Research Program through the National Research Foundation of Korea (NRF) (RS‐2024‐00345742).

## Supporting information



Supporting information

## Data Availability

The data used in this study are available from the corresponding author upon reasonable request.

## References

[1] S. Hong , S. H. Baek , M. K. P. Lai , T. V. Arumugam , and D. G. Jo , “Aging‐associated Sensory Decline and Alzheimer's Disease, ” Mol Neurodegener 19, no. 1 (2024): 93. 39633396 10.1186/s13024-024-00776-y PMC11616278

[2] Estimation of the Global Prevalence of Dementia in 2019 and Forecasted Prevalence in 2050: An Analysis for the Global Burden of Disease Study 2019. Lancet Public Health 2022;7(2):e105–e125. 34998485 10.1016/S2468-2667(21)00249-8 PMC8810394

[3] K. B. Rajan , J. Weuve , L. L. Barnes , E. A. McAninch , R. S. Wilson , and D. A. Evans , “Population Estimate of People With Clinical Alzheimer's Disease and Mild Cognitive Impairment in the United States (2020‐2060), ” Alzheimers Dement 17, no. 12 (2021): 1966–1975. 34043283 10.1002/alz.12362 PMC9013315

[4] J. Zissimopoulos , E. Crimmins , and P. St Clair , “The Value of Delaying Alzheimer's Disease Onset, ” Forum Health Econ Policy 18, no. 1 (2014): 25–39. 27134606 10.1515/fhep-2014-0013 PMC4851168

[5] J. Wang , B. J. Gu , C. L. Masters , and Y. J. Wang , “A Systemic View of Alzheimer Disease—insights From Amyloid‐beta Metabolism Beyond the Brain, ” Nature reviews Neurology 13, no. 10 (2017): 612–623. 28960209 10.1038/nrneurol.2017.111

[6] D. S. Knopman , H. Amieva , R. C. Petersen , et al., “Alzheimer Disease, ” Nature reviews Disease primers 7, no. 1 (2021): 33. 10.1038/s41572-021-00269-y PMC8574196 33986301

[7] Y. Cho , H. G. Bae , E. Okun , T. V. Arumugam , and D. G. Jo , “Physiology and Pharmacology of Amyloid Precursor Protein, ” Pharmacology & Therapeutics 235 (2022): 108122. 35114285 10.1016/j.pharmthera.2022.108122

[8] Y. L. Cheng , Y. Choi , C. G. Sobey , T. V. Arumugam , and D. G. Jo , “Emerging Roles of the γ‐secretase‐notch Axis in Inflammation, ” Pharmacology & Therapeutics 147 (2015): 80–90. 25448038 10.1016/j.pharmthera.2014.11.005

[9] D. M. Holtzman , J. C. Morris , and A. M. Goate , “Alzheimer's Disease: The Challenge of the Second Century, ” Science Translational Medicine 3, no. 77 (2011): 77sr1–77sr1. 21471435 10.1126/scitranslmed.3002369 PMC3130546

[10] H. Hampel , S. Lista , E. Vanmechelen , et al., “β‐Secretase1 Biological Markers for Alzheimer's Disease: State‐of‐art of Validation and Qualification, ” Alzheimers Res Ther 12, no. 1 (2020): 130. 33066807 10.1186/s13195-020-00686-3 PMC7566058

[11] Y. Luo , B. Bolon , S. Kahn , et al., “Mice Deficient in BACE1, the Alzheimer's Beta‐secretase, Have Normal Phenotype and Abolished Beta‐amyloid Generation, ” Nature Neuroscience 4, no. 3 (2001): 231–232. 11224535 10.1038/85059

[12] R. Vassar , B. D. Bennett , S. Babu‐Khan , et al., “Beta‐secretase Cleavage of Alzheimer's Amyloid Precursor Protein by the Transmembrane Aspartic Protease BACE, ” Science 286, no. 5440 (1999): 735–741. 10531052 10.1126/science.286.5440.735

[13] H. N. Woo , S. H. Baik , J. S. Park , et al., “Secretases as Therapeutic Targets for Alzheimer's Disease, ” Biochemical and Biophysical Research Communications 404, no. 1 (2011): 10–15. 21130746 10.1016/j.bbrc.2010.11.132

[14] M. Willem , A. N. Garratt , B. Novak , et al., “Control of Peripheral Nerve Myelination by the Beta‐secretase BACE1, ” Science 314, no. 5799 (2006): 664–666. 16990514 10.1126/science.1132341

[15] M. H. Ou‐Yang , J. E. Kurz , T. Nomura , et al., “Axonal Organization Defects in the Hippocampus of Adult Conditional BACE1 Knockout Mice, ” Science Translational Medicine 10, no. 459 (2018). 10.1126/scitranslmed.aao5620 PMC11017370 30232227

[16] G. Bahn , J. S. Park , U. J. Yun , et al., “NRF2/ARE Pathway Negatively Regulates BACE1 Expression and Ameliorates Cognitive Deficits in Mouse Alzheimer's Models, ” PNAS 116, no. 25 (2019): 12516–12523. 31164420 10.1073/pnas.1819541116 PMC6589670

[17] G. Bahn and D. G. Jo , “Therapeutic Approaches to Alzheimer's Disease through Modulation of NRF2, ” Neuromolecular Med 21, no. 1 (2019): 1–11. 30617737 10.1007/s12017-018-08523-5

[18] S. H. Baek , S. Hong , E. Kim , et al., “A Novel RAGE Modulator Induces Soluble RAGE to Reduce BACE1 Expression in Alzheimer's Disease, ” Adv Sci (Weinh) 12, no. 8 (2025): e2407812. 39755927 10.1002/advs.202407812 PMC11848596

[19] G. Zuliani , M. Ranzini , G. Guerra , et al., “Plasma Cytokines Profile in Older Subjects With Late Onset Alzheimer's Disease or Vascular Dementia, ” Journal of Psychiatric Research 41, no. 8 (2007): 686–693. 16600299 10.1016/j.jpsychires.2006.02.008

[20] M. T. Heneka , M. P. Kummer , A. Stutz , et al., “NLRP3 is Activated in Alzheimer's Disease and Contributes to Pathology in APP/PS1 Mice, ” Nature 493, no. 7434 (2013): 674–678. 23254930 10.1038/nature11729 PMC3812809

[21] C. K. Glass , K. Saijo , B. Winner , M. C. Marchetto , and F. H. Gage , “Mechanisms Underlying Inflammation in Neurodegeneration, ” Cell 140, no. 6 (2010): 918–934. 20303880 10.1016/j.cell.2010.02.016 PMC2873093

[22] B. Cameron and G. E. Landreth , “Inflammation, Microglia, and Alzheimer's Disease, ” Neurobiology of Disease 37, no. 3 (2010): 503–509. 19833208 10.1016/j.nbd.2009.10.006 PMC2823849

[23] C. Dempsey , A. Rubio Araiz , K. J. Bryson , et al., “Inhibiting the NLRP3 Inflammasome With MCC950 Promotes Non‐phlogistic Clearance of Amyloid‐β and Cognitive Function in APP/PS1 Mice, ” Brain, Behavior, and Immunity 61 (2017): 306–316. 28003153 10.1016/j.bbi.2016.12.014

[24] S. C. McQuown , R. M. Barrett , D. P. Matheos , et al., “HDAC3 is a Critical Negative Regulator of Long‐term Memory Formation, ” Journal of Neuroscience 31, no. 2 (2011): 764–774. 21228185 10.1523/JNEUROSCI.5052-10.2011 PMC3160172

[25] J. S. Guan , S. J. Haggarty , E. Giacometti , et al., “HDAC2 negatively Regulates Memory Formation and Synaptic Plasticity, ” Nature 459, no. 7243 (2009): 55–60. 19424149 10.1038/nature07925 PMC3498958

[26] J. S. Kim , J. H. Jun , J. Lee , et al., “HDAC6 mediates NLRP3 Inflammasome Activation in the Pathogenesis of Diabetic Retinopathy, ” Metabolism 164 (2025): 156108. 39689826 10.1016/j.metabol.2024.156108

[27] M. Gu , B. Ren , Y. Fang , et al., “Epigenetic Regulation in Cancer, ” MedComm 5, no. 2 (2024): e495. 38374872 10.1002/mco2.495 PMC10876210

[28] H. Ding , P. J. Dolan , and G. V. Johnson , “Histone Deacetylase 6 Interacts With the Microtubule‐associated Protein Tau, ” Journal of Neurochemistry 106, no. 5 (2008): 2119–2130. 18636984 10.1111/j.1471-4159.2008.05564.x PMC2574575

[29] P. Bai , P. Mondal , F. A. Bagdasarian , et al., “Development of a Potential PET Probe for HDAC6 Imaging in Alzheimer's Disease, ” Acta Pharm Sin B 12, no. 10 (2022): 3891–3904. 36213537 10.1016/j.apsb.2022.05.017 PMC9532562

[30] M. Zhang , W. Wang , Q. Ye , et al., “Histone Deacetylase Inhibitors VPA and WT161 Ameliorate the Pathological Features and Cognitive Impairments of the APP/PS1 Alzheimer's disease Mouse Model by Regulating the Expression of APP Secretases, ” Alzheimers Res Ther 16, no. 1 (2024): 15. 38245771 10.1186/s13195-024-01384-0 PMC10799458

[31] N. Govindarajan , P. Rao , S. Burkhardt , et al., “Reducing HDAC6 Ameliorates Cognitive Deficits in a Mouse Model for Alzheimer's disease, ” EMBO Molecular Medicine 5, no. 1 (2013): 52–63. 23184605 10.1002/emmm.201201923 PMC3569653

[32] V. G. Magupalli , R. Negro , Y. Tian , et al., “HDAC6 mediates an Aggresome‐Like Mechanism for NLRP3 and Pyrin Inflammasome Activation, ” Science 369, no. 6510 (2020). 10.1126/science.aas8995 PMC7814939 32943500

[33] J. Bockstiegel , S. L. Wurnig , J. Engelhardt , J. Enns , F. K. Hansen , and G. Weindl , “Pharmacological Inhibition of HDAC6 Suppresses NLRP3 Inflammasome‐mediated IL‐1β Release, ” Biochemical Pharmacology 215 (2023): 115693. 37481141 10.1016/j.bcp.2023.115693

[34] Y. Peng , J. Yu , F. Liu , et al., “Accumulation of TOX High Mobility Group Box family Member 3 Promotes the Oncogenesis and Development of Hepatocellular Carcinoma Through the MAPK Signaling Pathway, ” MedComm 5, no. 3 (2024): e510. 38463397 10.1002/mco2.510 PMC10924639

[35] E. K. Kim and E. J. Choi , “Pathological Roles of MAPK Signaling Pathways in human Diseases, ” Biochimica Et Biophysica Acta 1802, no. 4 (2010): 396–405. 20079433 10.1016/j.bbadis.2009.12.009

[36] C. Ploia , X. Antoniou , A. Sclip , et al., “JNK Plays a Key Role in Tau Hyperphosphorylation in Alzheimer's disease Models, ” Journal of Alzheimer's Disease 26, no. 2 (2011): 315–329. 10.3233/JAD-2011-110320 21628793

[37] X. Zhu , C. A. Rottkamp , H. Boux , A. Takeda , G. Perry , and M. A. Smith , “Activation of p38 Kinase Links Tau Phosphorylation, Oxidative Stress, and Cell Cycle‐related Events in Alzheimer disease, ” Journal of Neuropathology and Experimental Neurology 59, no. 10 (2000): 880–888. 11079778 10.1093/jnen/59.10.880

[38] S. Guise , D. Braguer , G. Carles , A. Delacourte , and C. Briand , “Hyperphosphorylation of Tau Is Mediated by ERK Activation During Anticancer Drug‐induced Apoptosis in Neuroblastoma Cells, ” Journal of Neuroscience Research 63, no. 3 (2001): 257–267. 11170175 10.1002/1097-4547(20010201)63:3<257::AID-JNR1019>3.0.CO;2-T

[39] B. Paudel , H. D. Bhattarai , H. K. Lee , H. Oh , H. W. Shin , and J. H. Yim , “Antibacterial Activities of Ramalin, Usnic Acid and Its Three Derivatives Isolated From the Antarctic Lichen Ramalina Terebrata, ” Z Naturforsch C J Biosci 65, no. 1‐2 (2010): 34–38. 20355318 10.1515/znc-2010-1-206

[40] B. Paudel , H. D. Bhattarai , H. Y. Koh , et al., “Ramalin, a Novel Nontoxic Antioxidant Compound From the Antarctic Lichen Ramalina Terebrata, ” Phytomedicine 18, no. 14 (2011): 1285–1290. 21802926 10.1016/j.phymed.2011.06.007

[41] S. S. Suh , T. K. Kim , J. E. Kim , et al., “Anticancer Activity of Ramalin, a Secondary Metabolite From the Antarctic Lichen Ramalina Terebrata, Against Colorectal Cancer Cells, ” Molecules (Basel, Switzerland) 22, no. 8 (2017). 10.3390/molecules22081361 PMC6152360 28817102

[42] T. K. Kim , J. M. Hong , K. H. Kim , et al., “Potential of Ramalin and Its Derivatives for the Treatment of Alzheimer's Disease, ” Molecules (Basel, Switzerland) 26, no. 21 (2021). 10.3390/molecules26216445 PMC8588271 34770857

[43] D. G. Jo , T. V. Arumugam , H. N. Woo , et al., “Evidence That Gamma‐secretase Mediates Oxidative Stress‐induced Beta‐secretase Expression in Alzheimer's Disease, ” Neurobiology of Aging 31, no. 6 (2010): 917–925. 18687504 10.1016/j.neurobiolaging.2008.07.003 PMC2858254

[44] A. R. Gwon , J. S. Park , T. V. Arumugam , et al., “Oxidative Lipid Modification of Nicastrin Enhances Amyloidogenic γ‐secretase Activity in Alzheimer's Disease, ” Aging Cell 11, no. 4 (2012): 559–568. 22404891 10.1111/j.1474-9726.2012.00817.x PMC4217088

[45] T. Gilon , O. Chomsky , and R. G. Kulka , “Degradation Signals for Ubiquitin System Proteolysis in Saccharomyces Cerevisiae, ” Embo Journal 17, no. 10 (1998): 2759–2766. 9582269 10.1093/emboj/17.10.2759 PMC1170616

[46] D. M. Kim , H. Kim , J. H. Yeon , J. H. Lee , and H. O. Park , “Identification of a Mitochondrial DNA Polymerase Affecting Cardiotoxicity of Sunitinib Using a Genome‐Wide Screening on S. pombe Deletion Library, ” Toxicological Sciences 149, no. 1 (2016): 4–14. 26385865 10.1093/toxsci/kfv210

[47] D. U. Kim , J. Hayles , D. Kim , et al., “Analysis of a Genome‐wide Set of Gene Deletions in the Fission Yeast Schizosaccharomyces Pombe, ” Nature Biotechnology 28, no. 6 (2010): 617–623. 10.1038/nbt.1628 PMC3962850 20473289

[48] M. T. Heneka , M. P. Kummer , and E. Latz , “Innate Immune Activation in Neurodegenerative Disease, ” Nature Reviews Immunology 14, no. 7 (2014): 463–477. 10.1038/nri3705 24962261

[49] S. W. Robinson , J. M. Bourgognon , J. G. Spiers , et al., “Nitric Oxide‐mediated Posttranslational Modifications Control Neurotransmitter Release by Modulating Complexin Farnesylation and Enhancing Its Clamping Ability, ” Plos Biology 16, no. 4 (2018): e2003611. 29630591 10.1371/journal.pbio.2003611 PMC5890968

[50] Y. Li , H. Wang , Y. Wang , et al., “Alterations in the Axon Initial Segment Plasticity Is Involved in Early Pathogenesis in Alzheimer's Disease, ” MedComm 5, no. 11 (2024): e768. 39415847 10.1002/mco2.768 PMC11473794

[51] S. H. Kim and T. A. Ryan , “CDK5 serves as a Major Control Point in Neurotransmitter Release, ” Neuron 67, no. 5 (2010): 797–809. 20826311 10.1016/j.neuron.2010.08.003 PMC2939042

[52] W. da Huang , B. T. Sherman , and R. A. Lempicki , “Systematic and Integrative Analysis of Large Gene Lists Using DAVID Bioinformatics Resources, ” Nature Protocols 4, no. 1 (2009): 44–57. 19131956 10.1038/nprot.2008.211

[53] B. T. Sherman , M. Hao , J. Qiu , et al., “DAVID: A Web Server for Functional Enrichment Analysis and Functional Annotation of Gene Lists (2021 update), ” Nucleic Acids Res 50, no. W1 (2022): W216–W221. 35325185 10.1093/nar/gkac194 PMC9252805

[54] M. Kanehisa and S. Goto , “KEGG: Kyoto encyclopedia of Genes and Genomes, ” Nucleic Acids Res 28, no. 1 (2000): 27–30. 10592173 10.1093/nar/28.1.27 PMC102409

[55] L. B. Yang , K. Lindholm , R. Yan , et al., “Elevated Beta‐secretase Expression and Enzymatic Activity Detected in Sporadic Alzheimer Disease, ” Nature Medicine 9, no. 1 (2003): 3–4. 10.1038/nm0103-3 12514700

[56] J. Han , J. H. Sul , J. Lee , et al., “Engineered Exosomes With a Photoinducible Protein Delivery System Enable CRISPR‐Cas‐based Epigenome Editing in Alzheimer's Disease, ” Science Translational Medicine 16, no. 759 (2024): eadi4830. 39110781 10.1126/scitranslmed.adi4830

[57] P. Wang , Z. Wang , and J. Liu , “Role of HDACs in Normal and Malignant Hematopoiesis, ” Molecular cancer 19, no. 1 (2020): 5. 31910827 10.1186/s12943-019-1127-7 PMC6945581

[58] J. H. Jun , J. S. Kim , L. F. Palomera , and D. G. Jo , “Dysregulation of Histone Deacetylases in Ocular Diseases, ” Arch Pharm Res 47, no. 1 (2024): 20–39. 38151648 10.1007/s12272-023-01482-x

[59] J. Park , H.‐J. Ha , E. S. Chung , et al., “O‐GlcNAcylation Ameliorates the Pathological Manifestations of Alzheimer's Disease by Inhibiting Necroptosis, ” Science Advances 7, no. 3 (2021): eabd3207. 33523877 10.1126/sciadv.abd3207 PMC7806231

[60] J. S. Kim , Y. Cho , J. Lee , et al., “N(5)‐((perfluorophenyl)amino)Glutamine Regulates BACE1, Tau Phosphorylation, Synaptic Function, and Neuroinflammation in Alzheimer's disease Models, ” Biosci Trends 19, no. 1 (2025): 102–115. 39864832 10.5582/bst.2024.01360

[61] S. H. Baek , S. J. Park , J. I. Jeong , et al., “Inhibition of Drp1 Ameliorates Synaptic Depression, Aβ Deposition, and Cognitive Impairment in an Alzheimer's Disease Model, ” Journal of Neuroscience 37, no. 20 (2017): 5099–5110. 28432138 10.1523/JNEUROSCI.2385-16.2017 PMC6596467

[62] S. Sarkar , E. Malovic , B. Plante , et al., “Rapid and Refined CD11b Magnetic Isolation of Primary Microglia With Enhanced Purity and Versatility, ” Journal of visualized experiments: JoVE no. 122 (2017). 10.3791/55364 PMC5555434 28447995

[63] J. R. Bae , W. Lee , Y. O. Jo , et al., “Distinct Synaptic Vesicle Recycling in Inhibitory Nerve Terminals Is Coordinated by SV2A, ” Progress in Neurobiology 194 (2020): 101879. 32615146 10.1016/j.pneurobio.2020.101879

[64] Y. Lee , S. Han , J. Lee , et al., “A Novel Multi‐target Compound Mitigates Amyloid Plaques, Synaptic Deficits, and Neuroinflammation in Alzheimer's disease Models, ” Arch Pharm Res 48, no. 7‐8 (2025): 745–764. 40770166 10.1007/s12272-025-01562-0

[65] T. K. Kim , Y. Cho , J. Kim , et al., “Synthesis and Evaluation of Chloride‐Substituted Ramalin Derivatives for Alzheimer's Disease Treatment, ” Molecules (Basel, Switzerland) 29, no. 15 (2024). 10.3390/molecules29153701 PMC11313798 39125105

[66] T. K. Kim , J. M. Hong , Y. Cho , et al., “Synthesis and Biological Evaluation of Novel Ramalin Derivatives as Multi‐Target Agents for Alzheimer's Disease, ” Molecules (Basel, Switzerland) 30, no. 9 (2025). 10.3390/molecules30092030 PMC12073177 40363835

[67] Y. Cho , J. Lee , J. S. Kim , et al., “RA‐PR058, a Novel Ramalin Derivative, Reduces BACE1 Expression and Phosphorylation of Tau in Alzheimer's disease Mouse Models, ” Anim Cells Syst (Seoul) 29, no. 1 (2025): 122–134. 39931645 10.1080/19768354.2025.2459649 PMC11809180

