# Supplementary material for: Ramalin Ameliorates Alzheimer's Disease Pathology by Targeting BACE1, HDAC6, and MAPK Pathways
Source: MedComm (2020). 2026 Jan 1;7(1):e70518. doi: 10.1002/mco2.70518 (PMC12757677; doi:10.1002/mco2.70518)
Supplement: Supplementary file 1 — Supporting information [file MCO2-7-e70518-s001.docx]

**Supplementary Information**

**Ramalin ameliorates Alzheimer's disease pathology by targeting BACE1, HDAC6, and MAPK pathways**

Yongeun Cho^1,#^, Jeongmi Lee^1,#^, Bo Youn Choi^1^, Jin-Ho Yun^1^, Sukmin Han^2^, Seung Hyun Baek^1^, Jinsu Park^1^, Yoonsuk Cho^1^, Hark Kyun Kim^1^, Eunae Kim^1^, Leon F Palomera^1^, Jeein Lim^1^, Yeji Jeon^1^, Jeonghyeong Im^1^, Ju-Mi Hong^3^, Tai Kyoung Kim^3^, Sung Hyun Kim^2,4^, Joung Han Yim^3,*^, Dong-Gyu Jo^1,5,6,7,*^

^1^School of Pharmacy, Sungkyunkwan University, Suwon 16419, Republic of Korea

^2^Department of Neuroscience, Graduate School, Kyung Hee University, Seoul 02447, Republic of Korea

^3^Division of Polar Life Sciences, Korea Polar Research Institute, Incheon 21990, Republic of Korea

^4^Department of Physiology, School of Medicine, Kyung Hee University, Seoul 02447, Republic of Korea

^5^Biomedical Institute for Convergence, Sungkyunkwan University, Suwon 16419, Republic of Korea

^6^Samsung Advanced Institute for Health Sciences & Technology (SAIHST), Sungkyunkwan University, Seoul 06351, Republic of Korea

^7^Institute of Quantum Biophysics, Sungkyunkwan University, Suwon 16419, Republic of Korea

* Correspondence: Joung Han Yim ([jhyim@kopri.re.kr](mailto:jhyim@kopri.re.kr)), and Dong-Gyu Jo ([jodg@skku.edu](mailto:jodg@skku.edu)).

^#^ Yongeun Cho and Jeongmi Lee contributed equally to this work.

**FIGURE S1 to S9**

**
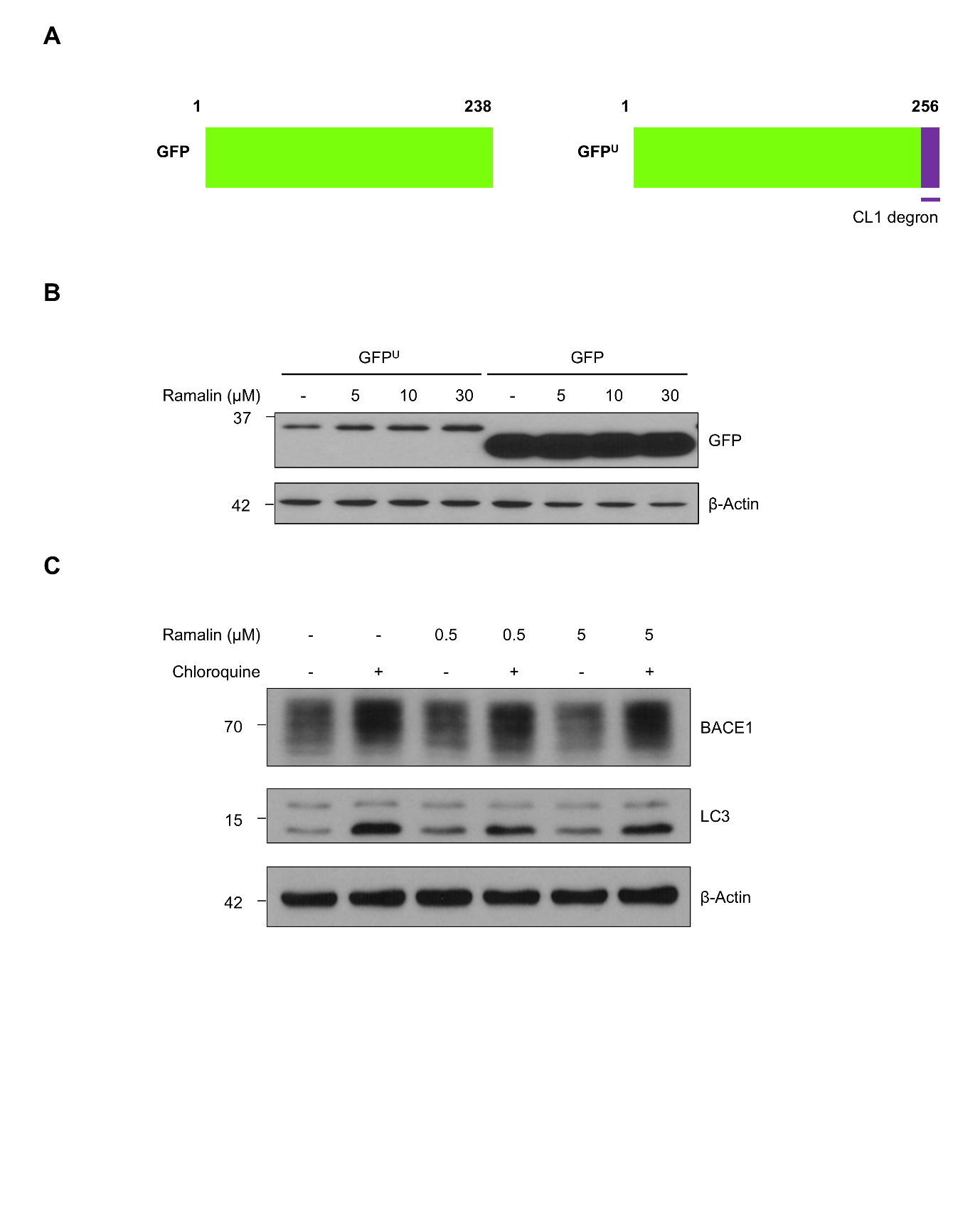
**

**FIGURE S1. Ramalin does not affect degradation of BACE1 through UPS or autophagy pathway.** (A) Schematic diagram of GFP or GFP^U^. CL1 degron signal sequence was attached to c-terminal of GFP. (B) Western blot analysis of GFP protein levels in HEK293T cells transfected with GFP or GFP^U^ plasmids and treated with 5, 10 and 30 μM of ramalin. (C) Western blot analysis of BACE1, LC3 protein levels in SH-SY5Y cells treated with 0.5, and 5 μM of ramalin and 10mM of chloroquine (CQ). CQ was used to inhibit autophagic degradation.


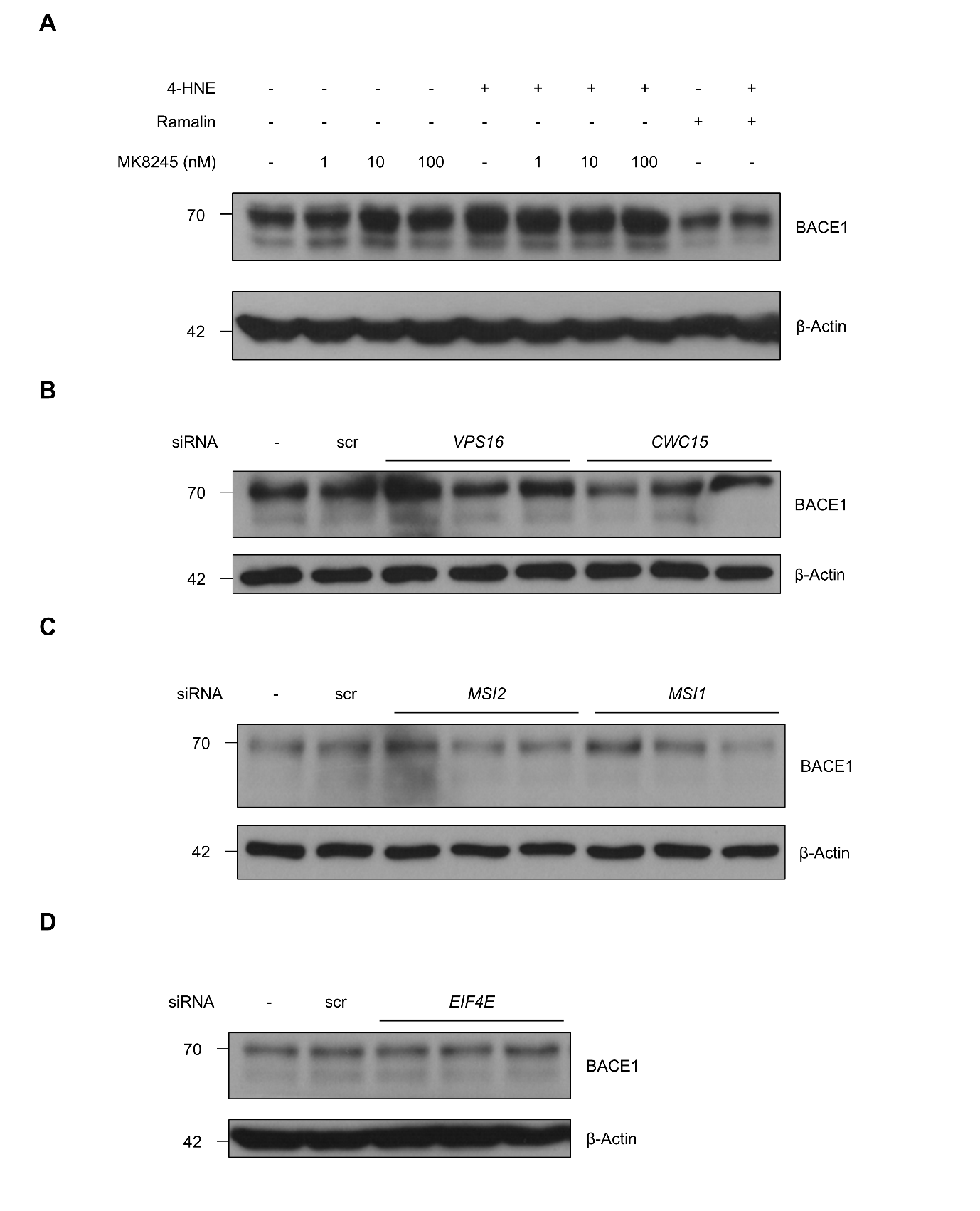


**FIGURE S2. Validation of predicted ramalin target genes obtained by *GPScreen*™.** (A) Western blot analysis of BACE1 protein levels in SH-SY5Y cells treated with 5 μM of 4-HNE, 1 μM of ramalin and 1, 10, 100 nM of MK8245, a potent SCD inhibitor. (B) Western blot analysis of BACE1 protein levels in HEK293T cells transfected with siVPS16 and siCWC15. (C) Western blot analysis of BACE1 protein levels in HEK293T cells transfected with siMSI1 and siMSI2. (D) Western blot analysis of BACE1 protein levels in HEK293T cells transfected with siEIF4E. Three different siRNAs were used to knock down each gene in (B-D). scr denotes scrambled siRNA.


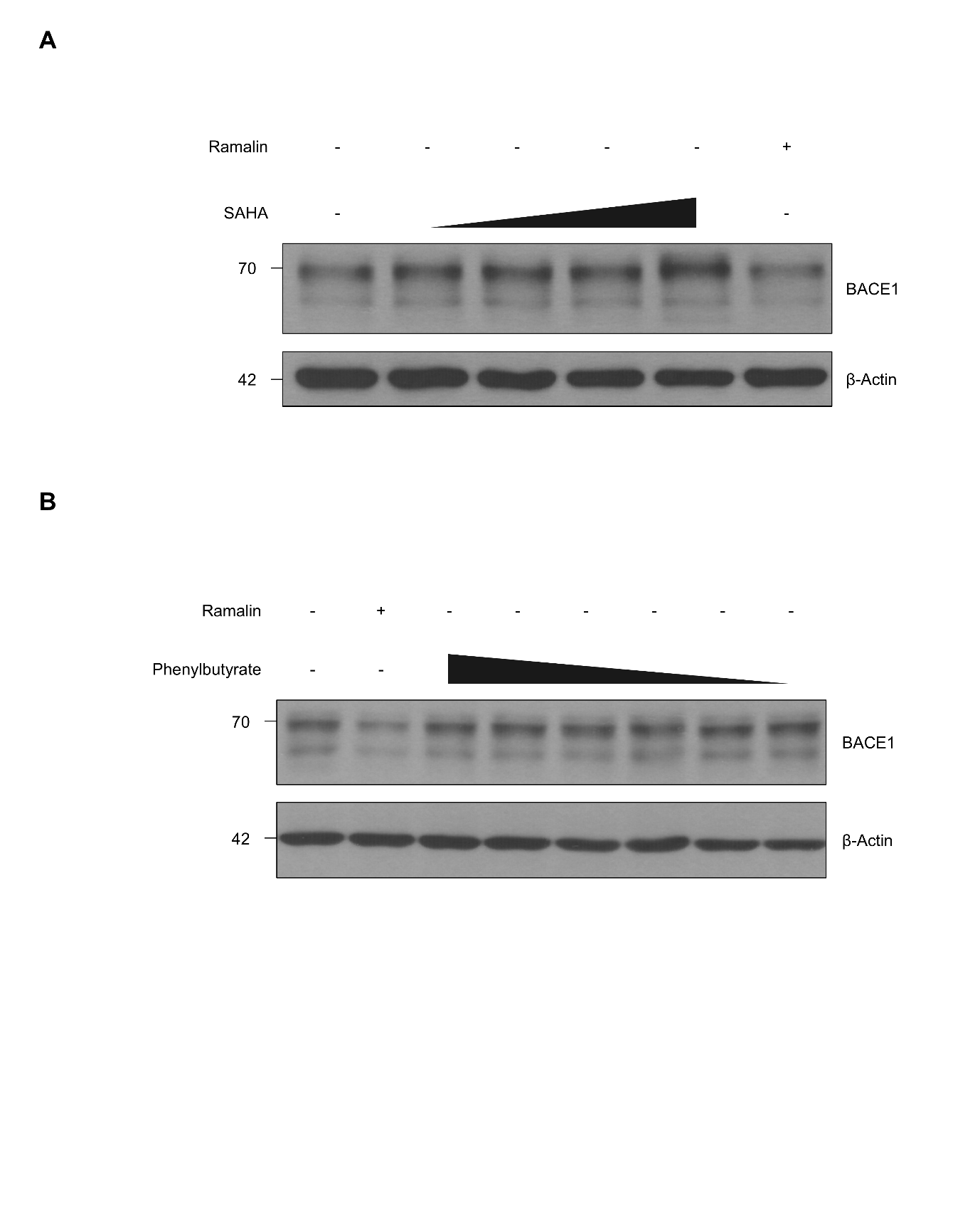


**FIGURE S3. Pan-HDAC inhibitors do not reduce BACE1 expression.** (A) Western blot analysis of BACE1 protein levels in SH-SY5Y cells treated with 1 μM of ramalin or 0.25, 10, 20 nM, and 5 μM of SAHA, a pan-HDAC inhibitor. (B) Western blot analysis of BACE1 protein levels in SH-SY5Y cells treated with 1 μM of ramalin or 1, 10, 20, 50, 100, and 400 μM of phenylbutyrate, a pan-HDAC inhibitor.


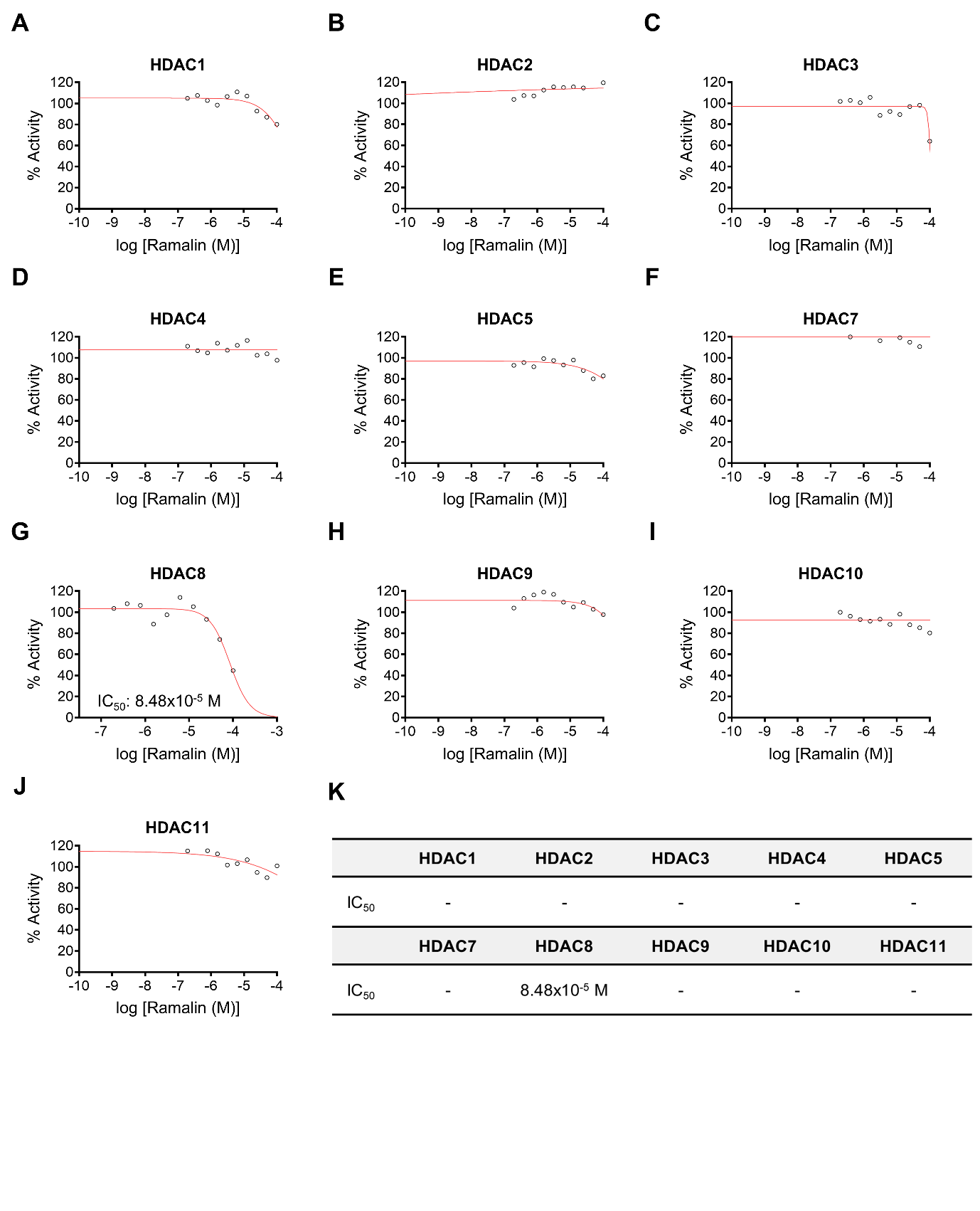


**FIGURE S4. Ramalin inhibits zinc dependent HDAC8.** (A-J) Zinc dependent HDACs inhibitory assay was conducted using ramalin in a dose-dependent manner. (K) IC_50_ values of ramalin on zinc dependent HDACs.

**
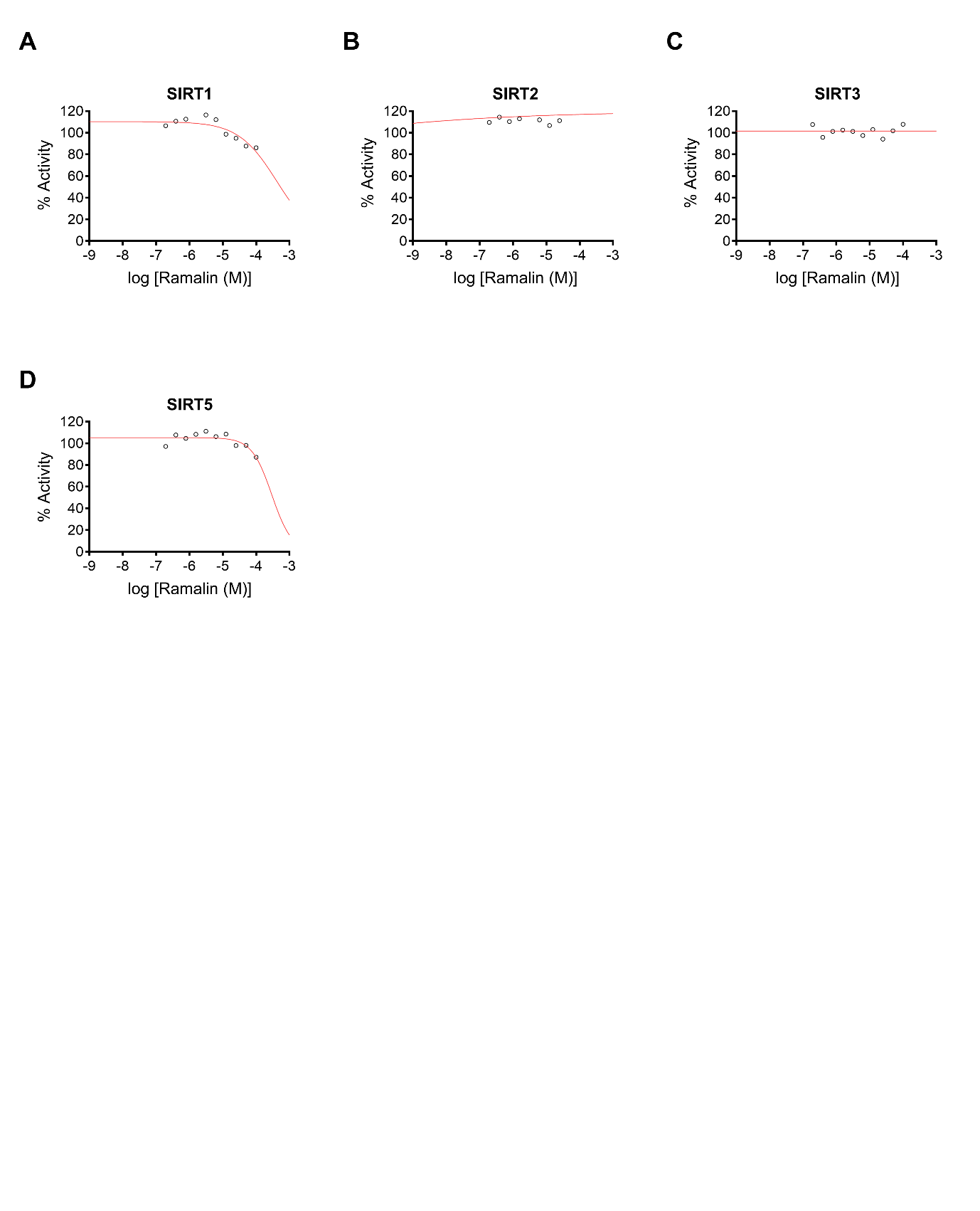
**

**FIGURE S5. Ramalin does not inhibit NAD^+^ dependent HDACs.** (A-D) NAD^+^ dependent HDACs inhibitory assay was conducted using ramalin in a dose-dependent manner.


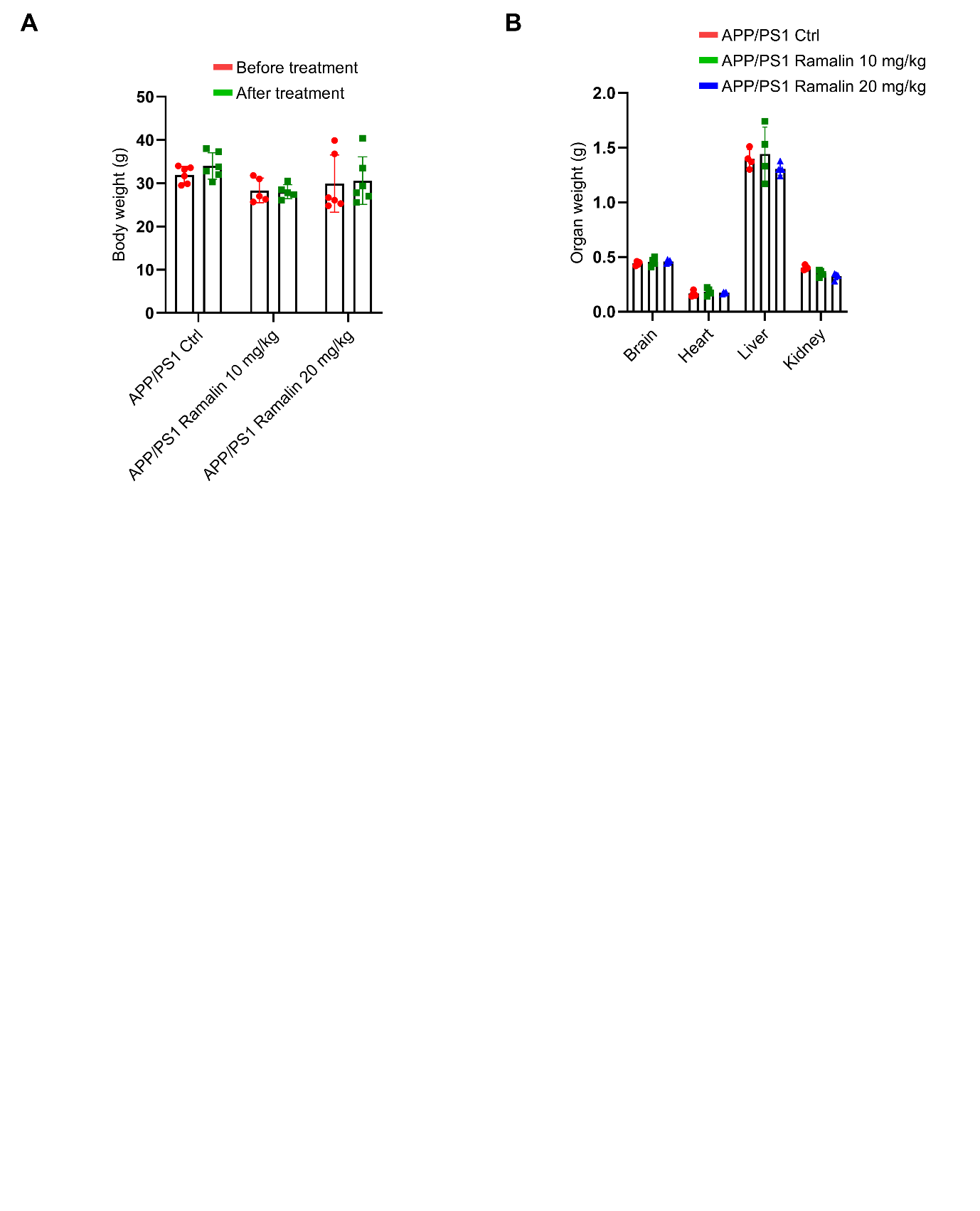


**FIGURE S6. Ramalin does not show side effects in APP/PS1 mice.** (A) Body weight of mice before/after ramalin treatment (APP/PS1 Ctrl; *n*=6, APP/PS1 Ramalin 10 mg/kg; *n*=5, APP/PS1 Ramalin 20 mg/kg; *n*=6). (B) Organ weight of mice before/after control or ramalin treatment (*n*=4 per group). Data are shown as mean ± SD in (A) and (B). Statistical significance was assessed using unpaired two-tailed t test in (A) and (B). ns; *p* > 0.05.


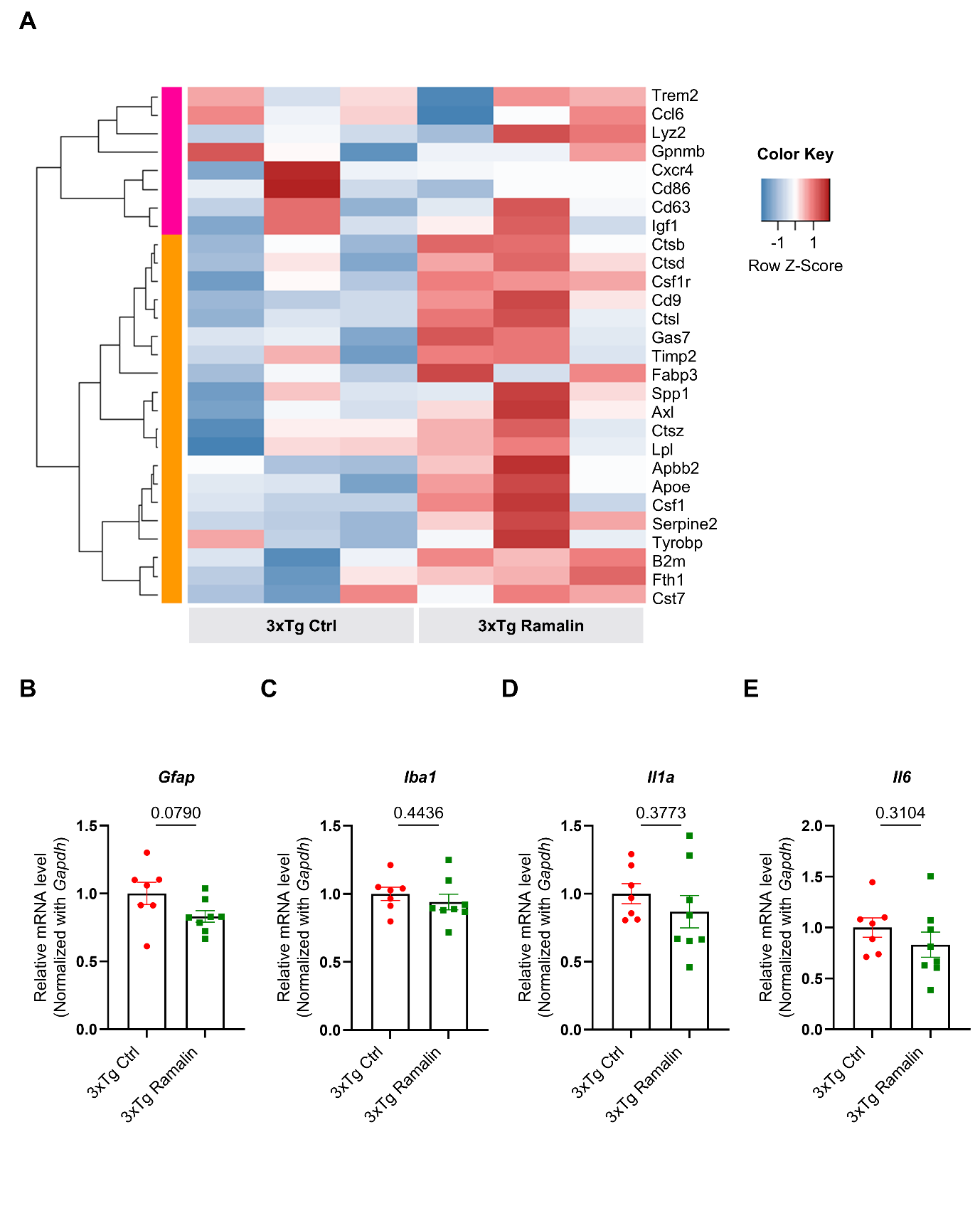


**FIGURE S7. Ramalin does not alter disease-associated microglia and glial/inflammatory marker.** (A) Heatmap of disease-associated microglia genes. (B-E) RT-qPCR analysis of *Gfap* (B), *Iba1* (C), *Il1a* (D), and *Il6* (E) mRNA levels in cortex of 3xTg Ctrl (*n*=7) and 3xTg Ramalin (*n*=8). Data are shown as mean ± SEM in (B), (C), (D), and (E). Statistical significance was assessed using unpaired two-tailed t test in (B), (C), (D), and (E).


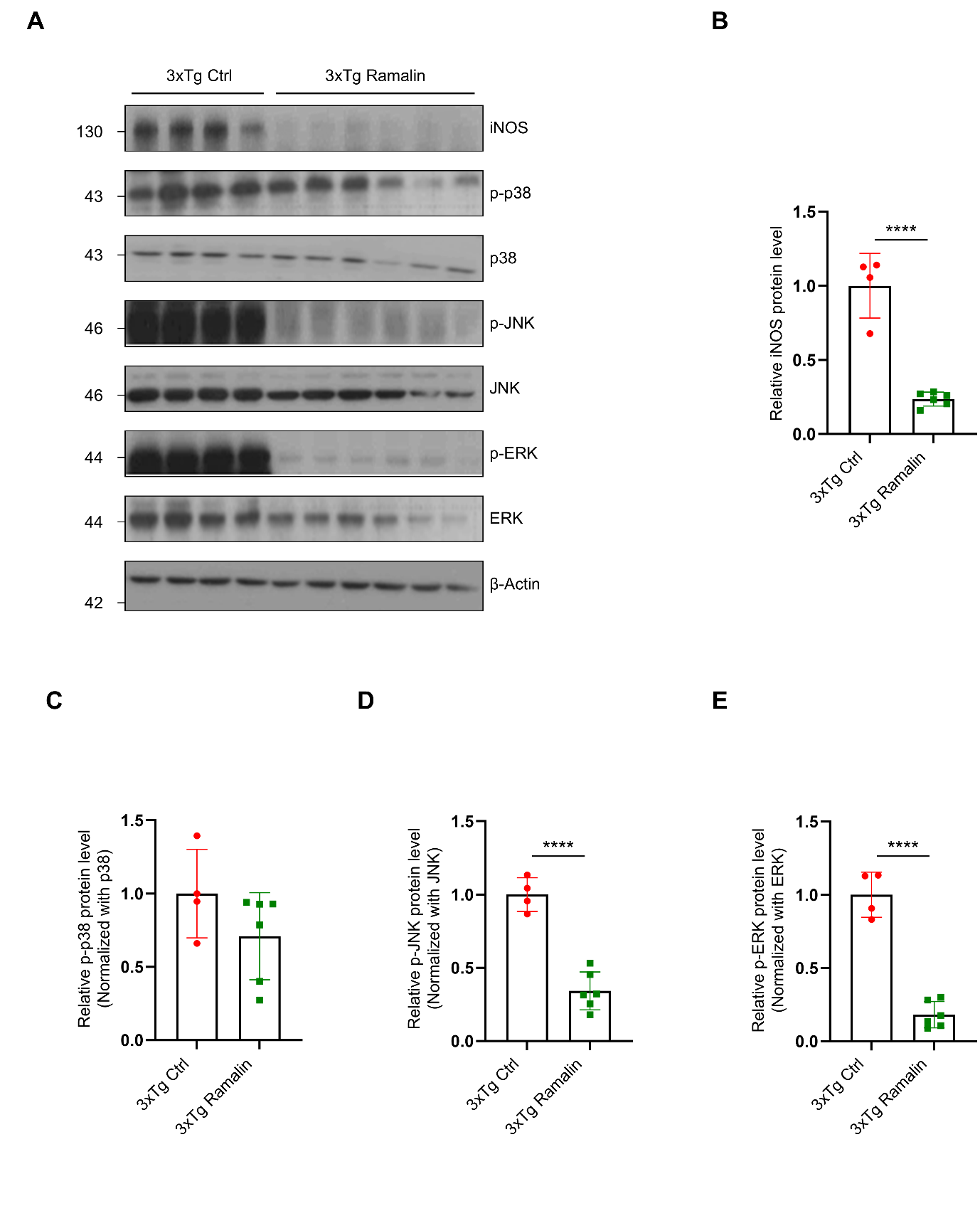


**FIGURE S8. Ramalin regulates inflammation and MAPK signaling pathway in 3xTg-AD mice at young age.** (A) Western blot analysis of iNOS, p-p38, p38, p-JNK, JNK, p-ERK and ERK in brain homogenates of young-aged 3xTg-AD mice (3xTg Ctrl; *n*=4, 3xTg Ramalin; *n*=6). (B-E) Quantification of iNOS in (B), p-p38/p38 in (C), p-JNK/JNK in (D) and p-ERK/ERK in (E) from (A). The expression level of iNOS was normalized to the expression level of β-Actin. Data are shown as mean ± SD in (B-E). Statistical significance was assessed using unpaired two-tailed t test in (B-E). *****p* < 0.0001.


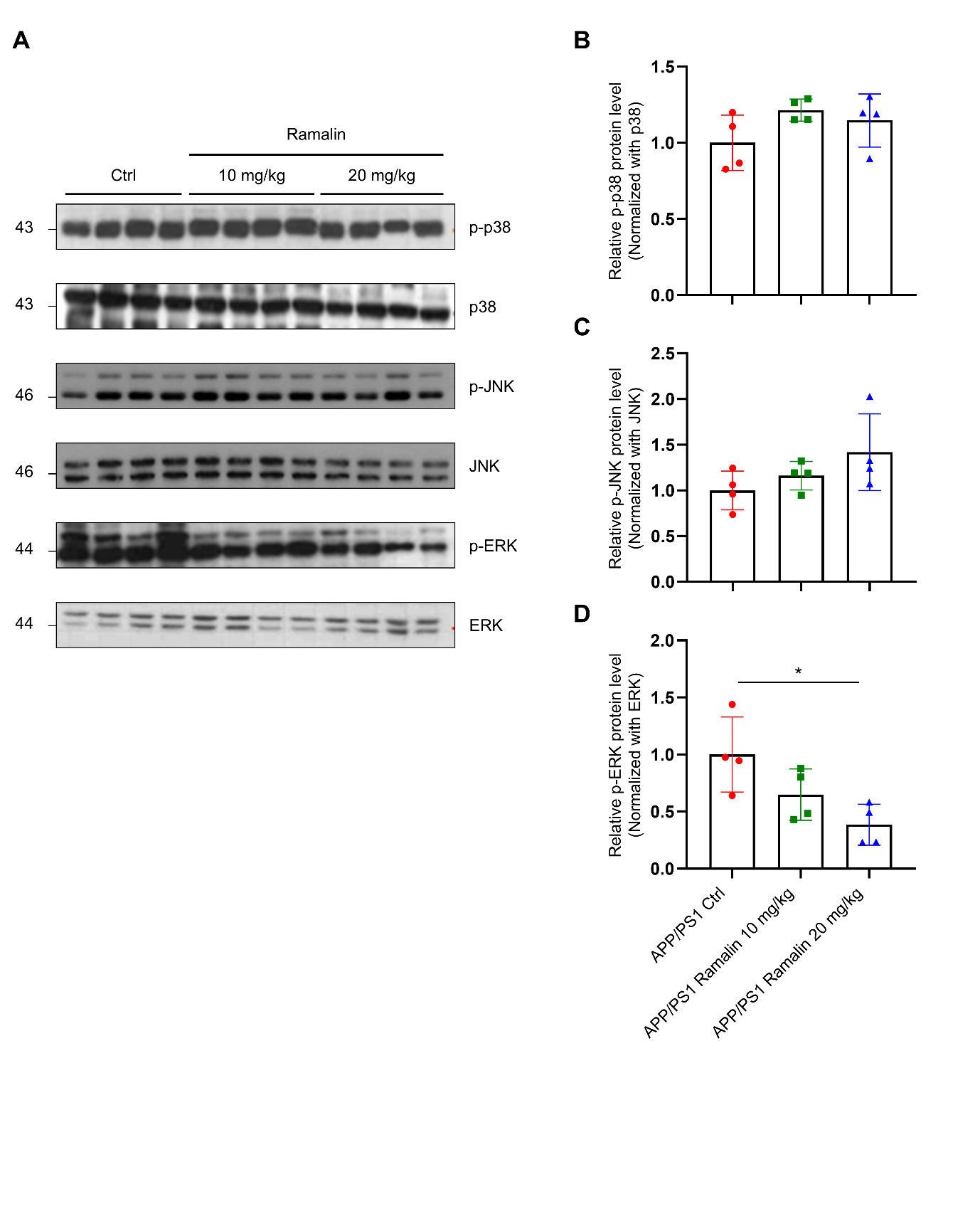


**FIGURE S9. Ramalin regulates MAPK signaling pathway in APP/PS1 brain homogenates.** (A) Western blot analysis of p-p38, p38, p-JNK, JNK, p-ERK and ERK in APP/PS1 brain homogenates (*n*=4 per group). (B-D) Quantification of p-p38/p38 in (B), p-JNK/JNK in (C), and p-ERK/ERK in (D) from (A). Data are shown as mean ± SD in (B-D). Statistical significance was assessed using unpaired two-tailed t test in (B-D). ns; *p* > 0.05; **p* < 0.05.
